# Bimetallic metal–organic frameworks and their derivatives

**DOI:** 10.1039/d0sc01432j

**Published:** 2020-04-28

**Authors:** Liyu Chen, Hao-Fan Wang, Caixia Li, Qiang Xu

**Affiliations:** AIST-Kyoto University Chemical Energy Materials Open Innovation Laboratory (ChEM-OIL), National Institute of Advanced Industrial Science and Technology (AIST) Yoshida, Sakyo-ku Kyoto 606-8501 Japan q.xu@aist.go.jp; School of Chemistry and Chemical Engineering, Yangzhou University Yangzhou 225002 China qxuchem@yzu.edu.cn

## Abstract

Bimetallic metal–organic frameworks (MOFs) have two different metal ions in the inorganic nodes. According to the metal distribution, the architecture of bimetallic MOFs can be classified into two main categories namely solid solution and core–shell structures. Various strategies have been developed to prepare bimetallic MOFs with controlled compositions and structures. Bimetallic MOFs show a synergistic effect and enhanced properties compared to their monometallic counterparts and have found many applications in the fields of gas adsorption, catalysis, energy storage and conversion, and luminescence sensing. Moreover, bimetallic MOFs can serve as excellent precursors/templates for the synthesis of functional nanomaterials with controlled sizes, compositions, and structures. Bimetallic MOF derivatives show exposed active sites, good stability and conductivity, enabling them to extend their applications to the catalysis of more challenging reactions and electrochemical energy storage and conversion. This review provides an overview of the significant advances in the development of bimetallic MOFs and their derivatives with special emphases on their preparation and applications.

## Abstract 2

This review summarizes the design and synthesis of bimetallic MOFs and their derivatives, with superior performance to their monometallic counterparts in many applications.

## Graphical abstract

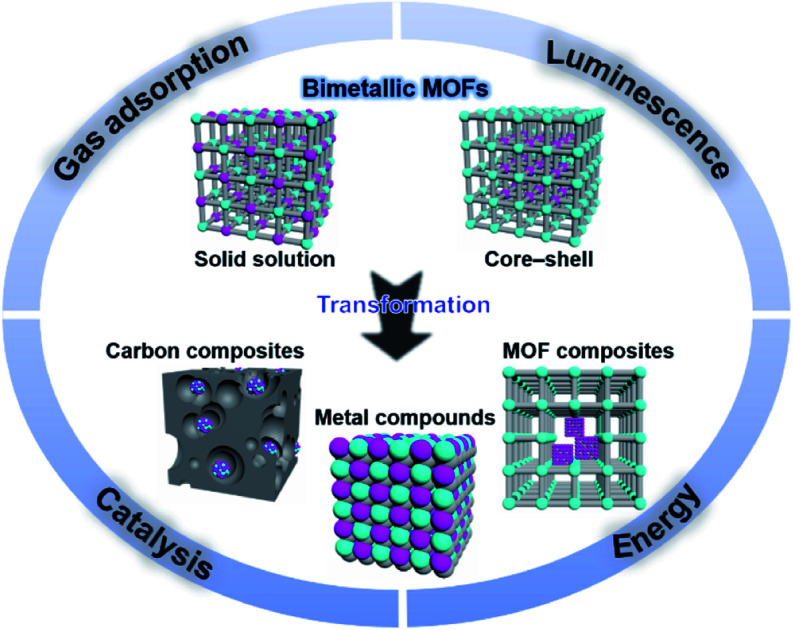

## 1. Introduction

Metal–organic frameworks (MOFs), or porous coordination polymers (PCPs), constructed from inorganic nodes with organic linkers, have emerged as a promising class of materials with high porosity, diverse composition and tuneable pore structures.^1,2^ These characteristics have attracted significant research interest in a variety of fields, such as gas adsorption and separation,^3–5^ catalysis,^6–8^ luminescence,^9^ sensing,^10^ biomedicine,^11^ and energy.^12–14^

In order to enhance the catalytic, electronic, and luminescence properties of MOFs, it has been proposed to incorporate second metal ions into the nodes of frameworks for the preparation of bimetallic MOFs.^15,16^ The partial substitution by second metal ions in the inorganic nodes or secondary-building units (SBUs) in the framework will allow the bimetallic system to show synergistic effects. In bimetallic MOFs, the proportions of the metals can be adjusted or even controlled, offering the possibility to tune the physicochemical properties of bimetallic MOFs.^17,18^

**Fig. 1:**
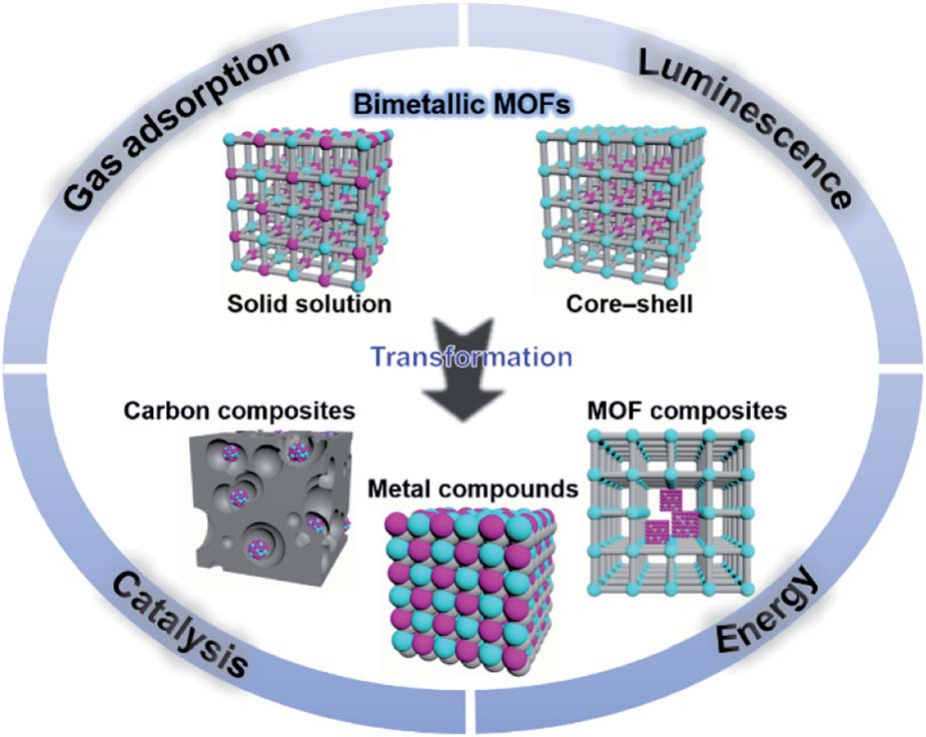
Schematic organization of the main contents including bimetallic MOFs and their derivatives, and some major applications.

According to the distribution of metal ions, bimetal MOFs can adopt “solid solution” or “core–shell” structures (Fig. 1).^19^ In solid solution bimetallic MOFs, the metals show delocalized or even homogeneous distributions through the whole crystal. Solid solution bimetallic MOFs can be synthesized by direct synthesis, post-synthetic modification and template synthesis. In core–shell bimetallic MOFs, the MOF shell is chemically different from the MOF core while they are integrated into a single architecture. Core–shell bimetallic MOFs can be synthesized through seed-induced growth of a MOF onto the surface of another, post-synthetic exchange of metal ions in the MOF surface, or one-pot synthesis. In this review, we mainly discuss the bimetallic MOFs in which both of metal ions serve as SBUs of MOFs. Bimetallic systems of immobilization of metal ions, metal nanoparticles (NPs), and metal complexes on the inorganic nodes,^20,21^ ligands,^22–24^ or inside MOF cavities^25–27^ as guests are outside of the scope of this review.^28,29^

Bimetallic MOFs with tuneable compositions and structures have shown enhanced properties compared to their monometallic counterparts, exhibiting superior performance in many applications, including gas adsorption, catalysis, energy storage and conversion, and luminescence sensing.^15,30–32^

Moreover, bimetallic MOFs can be used as precursors/templates for the synthesis of a variety of nanostructured materials, including carbon composites, metal compounds, and MOF composites (Fig. 1).^33,34^ By selecting appropriate bimetallic MOFs and controlling the post-treatment process (*e.g.*, pyrolysis, hydrolysis, and hydrothermal treatment), the compositions and structures of the afforded nanomaterials can be controlled. Bimetallic MOF-derived nanomaterials exhibit exposed active sites and high stability and conductivity, which can benefit their applications in the catalysis of more challenging reactions under harsh conditions and electrochemical energy storage and conversion.

**Fig. 2:**
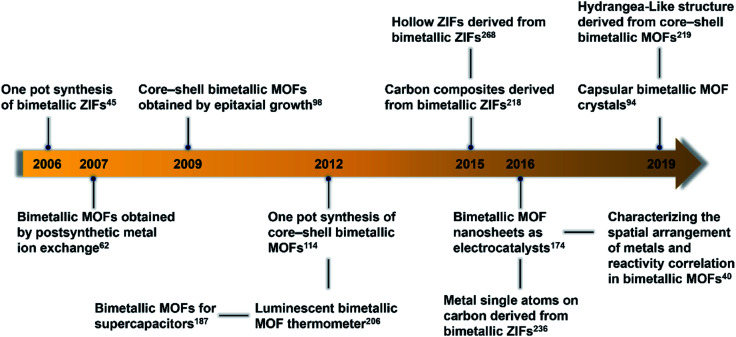
Timeline of important breakthroughs regarding bimetallic MOFs and their derivatives.

This review provides an overall picture of the significant advances of bimetallic MOFs (Fig. 2). Synthetic strategies of bimetallic MOFs and their derivatives are summarized. The applications of bimetallic MOFs and their derivatives in various fields including gas adsorption, catalysis, energy storage and conversion, and luminescence sensing are also presented. This review is expected to inspire more efforts in the development of bimetallic MOFs and their derivatives for a variety of applications.

## 2. Synthesis of bimetallic MOFs

Many strategies have been developed for the synthesis of bimetallic MOFs. This section focuses on the synthetic strategies of both solid solution and core–shell bimetallic MOFs. For the obtained bimetallic MOFs, using appropriate physicochemical techniques is essential for characterizing the compositions, distributions, and structures of the bimetallic MOFs. X-ray diffraction (XRD) and energy-dispersive X-ray (EDX) mapping can be combined to identify the crystalline phases of MOFs. In accordance with Vegard's law, bimetallic MOFs show diffraction peak positions between those of monometallic MOFs.^35,36^ Atomic absorption spectroscopy (AAS), inductively coupled plasma (ICP), EDX mapping and aerosol time-of-flight mass spectrometry (ATOFMS) can be coupled to calculate the global and local metal concentrations and distributions.^37,38^ X-ray absorption techniques, such as X-ray photoelectron spectroscopy (XPS) and X-ray absorption fine structure (XAFS), can determine the location of metals (present as nodes, bonded to the nodes, or present in the pores) in bimetallic MOFs.^39^ Extended X-ray absorption fine structure spectroscopy (EXAFS), neutron powder diffraction (NPD) and XPS can determine the spatial arrangements of metals in the SBUs of bimetallic MOFs.^40–42^ Different techniques should be combined for the accurate characterization of the synthesized bimetallic MOFs.

### 2.1 Solid solution bimetallic MOFs

**Fig. 3:**
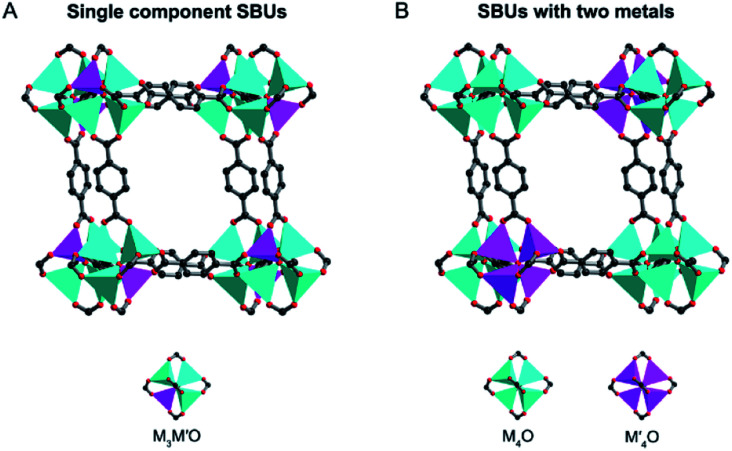
Two spatial arrangements of metals in solid solution bimetallic MOFs and their SBUs.

The term “solid solution” is used somewhat lightly, as in some cases a completely homogeneous distribution of metals may not be present.^16,35,43^ In this review, we divide solid solution bimetallic MOFs into two categories based on the spatial arrangements of metals: (1) two different metals are in the same SBU and the mixed metal SBUs are present throughout the MOF structure (Fig. 3A) and (2) each SBU consists of the same kind of metal and the two different SBUs are well mixed in the MOF structure (Fig. 3B). Two metals with a similar ionic radius and coordination sphere are more likely to exist in the same SBUs.^44^ It has been demonstrated that metals mixed in the same SBU can show stronger synergistic interactions compared to metals in different SBUs.^40^ However, in many bimetallic MOFs, the specific arrangement of metals in the SBUs throughout the crystal structure remains undetermined. Nevertheless, we generally define that in solid solution bimetallic MOFs the two metals are well dispersed throughout the MOF crystal. In this section, we focus on the discussion of methodologies to prepare solid solution bimetallic MOFs.

#### 2.1.1 Direct synthesis

Solid solution bimetallic MOFs can be directly synthesized by mixing all the metal salts during the solvothermal synthesis.^45–48^ Using two metal salts in a one-pot reaction does not guarantee the formation of a solid solution bimetallic MOF due to the varying MOF-forming kinetics of the two metal ions. To achieve a controlled incorporation, delicate control is required to avoid the formation of mixed MOF phases. Parameters including the solubility, reactivity and coordination sphere of metal ions and the pH of the reaction mixture have significant effects on the final molar ratio of the metal ions.

**Fig. 4:**
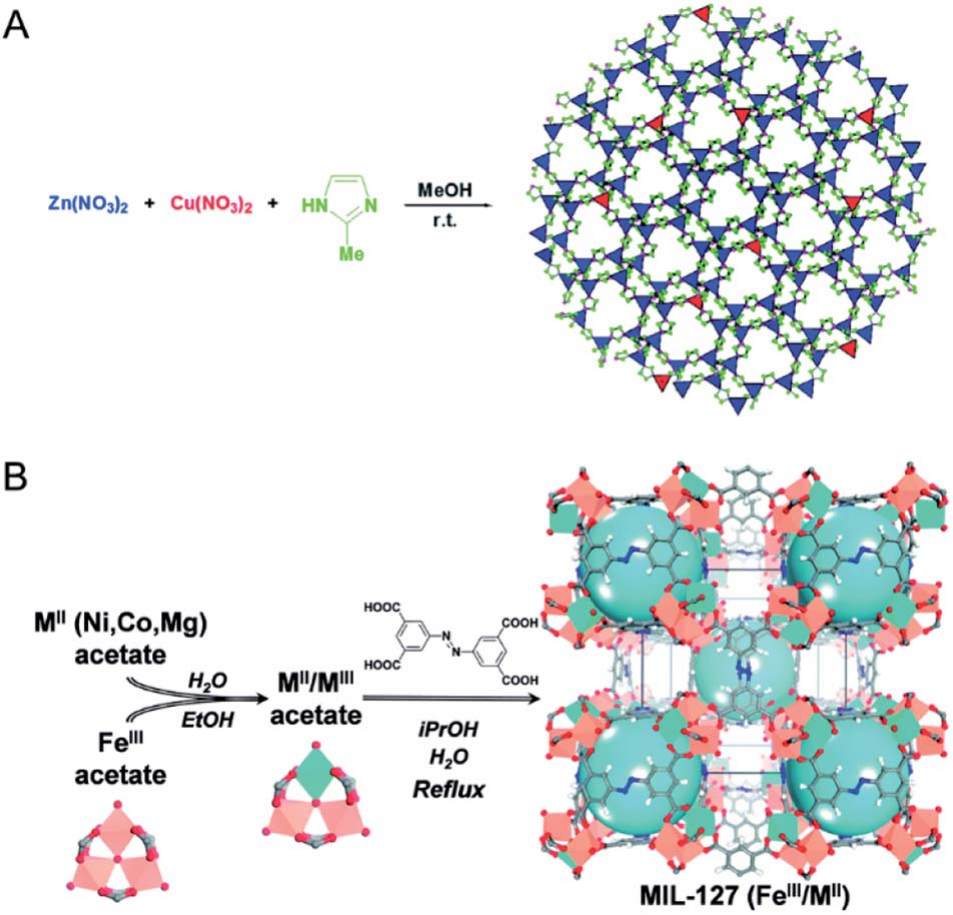
(A) Schematic representation of the synthesis of Cu-doped ZIF-8 crystals. Reproduced with permission from ref. 53. Copyright 2015, Royal Society of Chemistry. (B) Schematic representation of the building block approach to produce mixed-metal carboxylate MOFs such as MIL-127. From left to right, mixing iron acetate trimeric building blocks with M^II^ acetate compounds leads to neutral mixed-metal trimeric building blocks that are used to produce the titled MOF *via* acetate/ligand exchange. Reproduced with permission from ref. 60. Copyright 2015, Royal Society of Chemistry.

A variety of bimetallic MOFs have been successfully synthesized through adding second metal precursors in the recipe of monometallic MOFs, such as Co^2+^ doping into MOF-5 (IRMOF-1, Zn_4_O(BDC)_3_, BDC = terephthalate) for Co/MOF-5,^49^ Zn^2+^ doping into HKUST-1 (Cu_3_(BTC)_2_, BTC = 1,3,5-benzenetricarboxylate) for Zn/Cu-BTC,^50^ Co doping into MOF-74 (Zn_2_(DOT), DOT = dioxidoterephthalate) for Co/MOF-74,^51^ Cu^2+^ doping into ZIF-67 (Co(2-MeIm)_2_, 2-MeIm = 2-methylimidazole, ZIF stands for the zeolitic imidazolate framework) for Cu/ZIF-67,^52^ Cu into ZIF-8 (Zn(2-MeIm)_2_) for Cu/ZIF-8 (Fig. 4A),^53^ Mg^2+^ doping into MIL-101(Cr) (MIL stands for Materials Institute Lavoisier),^54^ MIL-53(Cr–Fe) [(M^III^OH)(BDC)(H_2_BDC)_*x*_],^55^ and Ce doping into UiO-66(Zr) (UiO stands for Universitetet i Oslo).^56^

Mixed-metal MOFs containing more than two kinds of metal ions have been achieved. Yaghi and co-workers use a one-pot reaction to make crystalline mixed-metal MOF-74 [M_2_(DOT)] of varying metal ions (up to 10 different metal ions) in the nodes.^57^ H_4_DOT and selected metal salts were dissolved in a solvent mixture of *N*,*N*-dimethylformamide (DMF), ethanol, and water, heated at 120 °C for 20–24 h, affording mixed-metal MOF-74 (MM-MOF-74) materials. It is worth mentioning that this approach allowed the incorporation of typical metal ions (*i.e.*, Ca, Sr, Ba, and Cd) in MM-MOF-74 from which a single-metal-containing MOF structure could not be made. XRD and SEM confirmed the pure phase of MM-MOF-74. Inductively coupled plasma-optical emission spectrometry (ICP-OES) demonstrated that MM-MOF-74 contained all metal ions added to the reaction mixture, while Ca, Sr, and Ba ions were less present because of their higher coordination number (8) than that of Mg, Mn, Fe, Co, Ni, Zn and Cd (6). EDX indicated that MM-MOF-74 crystals contained all the metals but had different distributions of the metals in each crystal.

To reach a higher degree of control over the synthesis of mixed-metal MOFs, the use of pre-defined SBUs is a promising approach to control metal stoichiometry.^58–61^ Feng, Bu and co-workers created a variety of In-M clusters as inorganic building blocks for fabricating five series of MOFs, CPM-18 M (M = Nd, Sm), CPM-19 M (M = Nd, Pr), CPM-20 (Co), CPM-21 M (M = Mn, Co, Cu), and CPM-23 (Mg).^58^ This work demonstrated the successful synthesis of bimetallic MOFs consisting of a p-block element (indium) and s-, d-, and f-block elements with differing nuclearities, metal-to-metal ratios, geometries, and charges. Serre and co-workers used oxocentered trimeric mixed iron(iii)/metal(ii) acetates (M = Co, Ni, Mg) as building blocks for the scalable and green preparation of mixed-metal MIL-127 (Fig. 4B).^60^ They first synthesized neutral mixed metal building blocks with the general formula Fe^III^_2_M^II^O(H_2_O)_2_[O_2_C-CH_3_]_6_·_*n*_H_2_O (with M = Co or Ni), which were then used as precursors for the synthesis of Fe/Co, Fe/Ni and Fe/Mg MIL-127 MOFs. EDX analysis indicated that the ratio of Fe(iii) to metal(ii) remained at *ca*. 2 to 1 in the obtained MOFs. A control experiment using a 2 to 1 ratio of Fe(iii) and metal(ii) salts under similar synthesis conditions led to either amorphous or recrystallized linkers due to the different acidity and metal–ligand bond lability between metal(iii) and metal(ii) cations.

#### 2.1.2 Post-synthetic modification

Bimetallic MOFs can be fabricated by postsynthetic metal ion exchange processes, which may not be yielded through direct synthesis.^62–64^ The coordination number and environment of the SBUs, the ionic radii and the valency of the metal ions, the MOF lattice and the solvent significantly affect the extent, rate, and reversibility of the exchange process.^65–68^ Metal ions in an SBU with higher coordination numbers or coordinating with terminal solvent species can undergo cation exchange.^69–72^ Cu^2+^ ions tend to replace most other second row transition metals (Zn^2+^, Cd^2+^, and Mn^2+^) due to their high electronegativity to form more covalent bonds with thermodynamic stability.^63,73,74^ Cd^2+^ and Pb^2+^ show a faster exchange rate than Cu^2+^, owing to their low electronegativity to form labile ionic bonds.^75–77^ MOFs with great lattice flexibility can allow the geometrical distortions of SBUs for cation exchange.^78^ Solvents with a small molecular size and relatively high ligand field strength (*e.g.*, methanol) can accelerate the exchange rate compared with larger solvents (DMF or dimethyl sulfoxide).^63,79^

Long and co-workers demonstrated the concept of cation exchange of MOFs by partially exchanging Mn^2+^ in Mn_3_[(Mn_4_Cl)_3_(BTT)_8_(CH_3_OH)_10_]_2_ (1-Mn^2+^; BTT = 1,3,5-benzenetristetrazolate) with selected cations.^62^ The MOFs were soaked in methanolic solutions of Fe^2+^, Co^2+^, Ni^2+^, Cu^2+^, Zn^2+^, Li^+^, and Cu^+^ salts at room temperature. The degree of cation exchange in 1-Mn^2+^ was determined by inductively coupled plasma-atomic absorption (ICP-AA). The 3 equiv. of Mn^2+^ in 1-Mn^2+^ could be replaced with Fe^2+^, Co^2+^, Ni^2+^, Cu^2+^, and Zn^2+^. However, the metathesis of Mn^2+^ with monovalent cations (Li^+^ or Cu^+^) showed incomplete exchange with Li^+^ or almost negligible substitution with Cu^+^ due to charge repulsion effects.

Kim and co-workers reported the complete and reversible exchange of metal ions in the SBUs of a microporous framework while maintaining the structural integrity of the framework and single crystallinity.^75^ The Cd^2+^ ions in MOF **1**, Cd_1.5_(H_3_O)_3_[(Cd_4_O)_3_(hett)_8_]·6H_2_O (hett = ethyl substituted truxene tricarboxylate), were exchanged with Pb^2+^ by immersing MOF **1** in aqueous Pb(NO_3_)_2_ solution. The monitoring of the ion exchange process by inductively coupled plasma atomic emission spectroscopy (ICP-AES) showed that 98% of Cd^2+^ was replaced by Pb^2+^ within 2 h and a complete exchange of Cd^2+^ with Pb^2+^ occurred in 2 days. A reverse exchange of Pb^2+^ with Cd^2+^ took a longer time with ∼50% exchange of Pb^2+^ in 1 day and a complete exchange in almost 3 weeks. Moreover, the Cd^2+^ ions of MOF **1** could be exchanged with trivalent lanthanide ions (Dy^3+^ and Nd^3+^) balanced by NO_3_^−^ ions without losing crystallinity.

Metal ion exchange has focused largely on MOFs that are not considered highly robust,^63,76^ while it is also observed on MOFs that are considered to possess high structural stability, such as ZIFs,^38^ UiO-66(Zr),^37,80^ and MILs.^81^

**Fig. 5:**
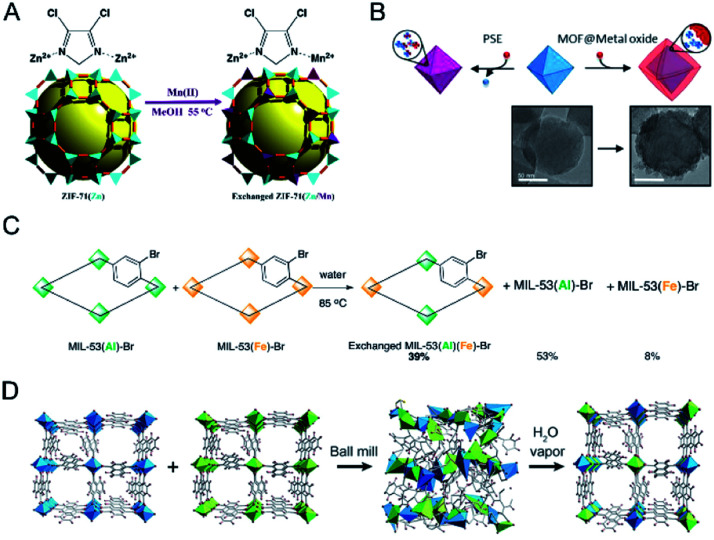
(A) Postsynthetic Mn(ii) exchange of ZIF-71. Reproduced with permission from ref. 38. Copyright 2013, American Chemical Society. (B) The addition of exogenous Ti^4+^ and Hf^4+^ to UiO-66 resulted in the formation of metal oxides on the surface of UiO-66 particles, giving a core–shell, MOF@metal oxide structure. Reproduced with permission from ref. 83. Copyright 2018, American Chemical Society. (C) Solid–solid postsynthetic cation exchange in MIL-53. Reproduced with permission from ref. 37. Copyright 2012, American Chemical Society. (D) A possible formation mechanism for the solid solution of Al-ndc and Ga-ndc by ball milling and vapor exposure processes. Reproduced with permission from ref. 85. Copyright 2017, Wiley-VCH.

Cohen and co-workers reported the synthesis of bimetallic ZIFs (Mn(ii)-based ZIFs) through a postsynthetic exchange approach (Fig. 5A).^38^ ZIF-71(Zn) was incubated in a solution of Mn(acetylacetonate)_2_ in MeOH to afford ZIF-71(Zn/Mn). Results showed that all of the ZIF-71 particles participated in the exchanged process with 12% of the tetrahedral Zn^2+^ centres exchanged for Mn^2+^. ZIF-8 could also exchange ∼10% of Zn(ii) centres for Mn(ii). Cohen and co-workers also demonstrated the metal exchange of Zr^4+^ in UiO-66(Zr) with Ti^4+^ and Hf^4+^.^37^ UiO-66(Zr) was exposed to DMF solutions of different Ti^4+^ salts, such as TiCp_2_Cl_2_, TiCl_4_(THF)_2_, or TiBr_4_ (Cp = *η*^5^-cyclopentadienyl, THF = tetrahydrofuran) for 5 days at 85 °C. The amount of exchanged Ti^4+^ depended on the metal salt used. TiCl_4_(THF)_2_ showed the highest substitution level (38 wt%), while TiBr_4_ showed the lowest level due to its reactivity and instability. UiO-66(Zr) allowed very little metal ion exchange with HfCl_4_ at room temperature. Even at elevated temperatures, only ∼20% of the UiO-66(Zr) particles incorporated Hf^4+^.

One should keep in mind that the postsynthetic exchange of metal ions cannot always allow the replacement of metal ions in the SBUs. The metal ions may graft onto the surface of the inorganic node at a linker vacancy site or deposit on the surface of MOFs in the form of nanoscale metal oxides (Fig. 5B).^82,83^ Different analytical methods should be combined to confirm the successful exchange of metal ions in the SBUs of MOFs after synthesis.

Moreover, metal exchange can also occur between two robust MOF solids. Cohen and co-workers demonstrated that robust MOFs would readily exchange their structural metal ions in a solid–solid postsynthetic exchange process (Fig. 5C).^37^ MIL-53(Al)–Br and MIL-53(Fe)–Br as dry solids were mixed and then incubated in water for 5 days at 85 °C. ATOFMS showed that 40% of the particles contained both metal ions [*i.e.*, MIL-53(Al/Fe)].

In addition, mechanical milling has also been demonstrated to be a powerful tool for metal ion exchange between two solid MOFs for the synthesis of solid solution bimetallic MOFs.^84–86^ Horike, Kitagawa and co-workers demonstrated that solvent-free mechanical milling of two MOF crystals could induce the dissolution of each metal ion into the amorphous solid state, which subsequently reconstructed to crystalline structures *via* vapor treatment (Fig. 5D).^85^ The authors prepared nine powder mixtures of Al-ndc and Ga-ndc (ndc = 1,4-naphthalenedicarboxylate) with different molar ratios from 1 : 9 to 9 : 1. The mixtures were treated by ball-milling, followed by exposure to saturated water for 3 days to form solid solution Al/Ga-ndc materials, as determined by XRD. This method could also be applied for the preparation of other solid solution MOFs, such as Zn/Mg-MOF-74, Zn/Co-ZIF-8, and Zn/Cd-ZIF-8.

#### 2.1.3 Template synthesis

**Fig. 6:**
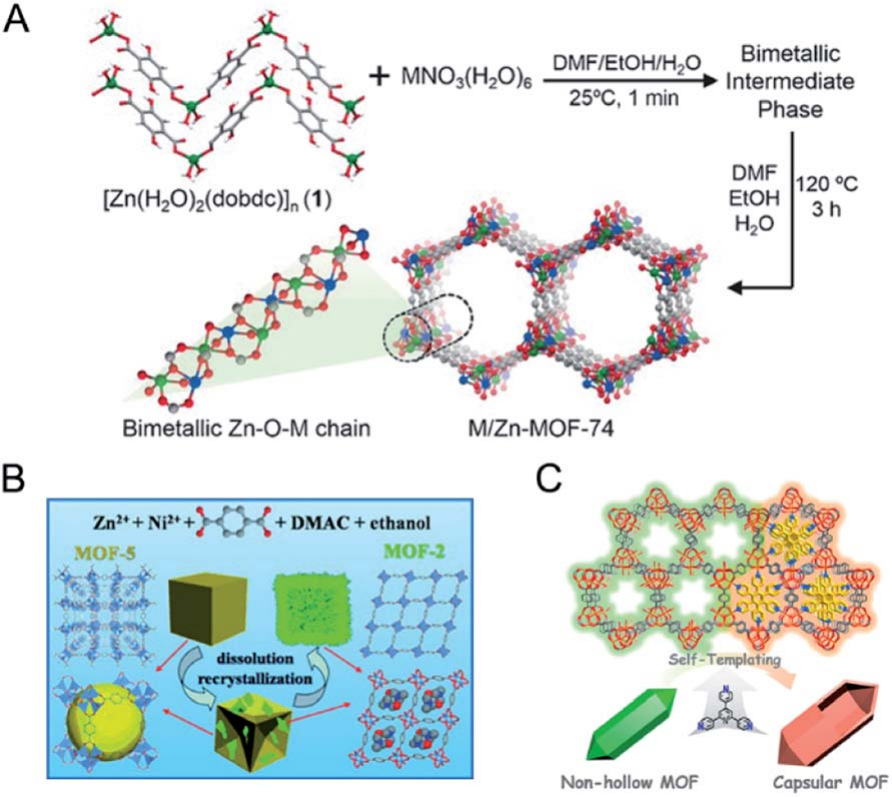
(A) Synthetic strategy for the heterogeneous phase preparation of bimetallic MOF-74 structures *via* a template-directed approach, in which a 1D metal–organic polymer with well-defined binding pockets was used as a structural template for the preparation of well-ordered bimetallic MOF-74s. Reproduced with permission from ref. 87. Copyright 2017, Wiley-VCH. (B) Schematic illustration of crystal-structure transformation of a MOF combined with its shape evolution. Reproduced with permission from ref. 93. Copyright 2014, Wiley-VCH. (C) Schematic illustration for the formation of capsular-MOF from FeNi-MIL-88B. Reproduced with permission from ref. 94. Copyright 2019, American Chemical Society.

Template methods have been employed for the synthesis of bimetallic MOFs for a good control over the arrangement of metal ions in MOFs. Coskun and co-workers employed a metal–organic polymer with well-defined binding sites for anchoring a secondary metal as a structural template and precursor for the synthesis of bimetallic MOF-74 s (Fig. 6A).^87^ 1D metal–organic polymer 1, [Zn(H_2_O)_2_(C_8_H_4_O_6_)]_*n*_, was synthesized by reacting 2,5-dihydroxy-1,6-benzenedicarboxylate with ZnSO_4_. The 1D polymer possessed well-defined binding sites (hydroxy and carbonyl) for the coordination of secondary metal ions (Mg^2+^ or Ni^2+^). The 1D polymer was suspended in a solution of the secondary metal precursors to get impregnated polymers, followed by transformation into 3D Zn/M-MOF-74 (M = Mg, Ni) under solvothermal conditions. The two different metal ions were homogeneously distributed in MOF-74 crystals with a constant molar ratio of 1 : 1 regardless of the initial metal concentrations in the reaction mixture.

In addition to achieving a better control of the metal arrangement in bimetallic MOFs, templated synthesis can also allow the construction of hollow bimetallic MOFs. Hollow MOFs can not only inherit the merits of MOFs, but also have additional advantages such as facile mass transport and rich active sites.^88^ Templated synthesis methods for the synthesis of hollow MOFs can be divided into the exterior-template method and self-template method. Exterior-template synthesis of hollow MOFs usually uses a sacrificial template to prefabricate a core–shell intermediate and subsequently removes the template.^89,90^ This exterior-template method has been applied for the synthesis of hollow bimetallic MOFs.^91^ The self-template method has also been reported for the synthesis of hollow bimetallic MOFs.^92–95^ The self-template method usually goes through a dissolution–regrowth process, leading to the formation of bimetallic MOFs with hollow structures. The self-template method does not require a further step to remove the templates, which is simple and facile. For example, Wang and co-workers reported the synthesis of hierarchical Zn/Ni-MOF-2 nanosheet assembled hollow nanocubes (NAHNs) transformed from Zn/Ni-MOF-5 *via* a dissolution and recrystallization process (Fig. 6B).^93^ H_2_BDC, Zn^2+^ and Ni^2+^ ions were dissolved in a mixed solvent of *N*,*N*-dimethylacetamide (DMAC) and ethanol. During the synthesis process, a high concentration of precursors led to a fast formation of cubic crystalline Zn/Ni-MOF-5, which was gradually etched and acted as the template for the growth of thermodynamically favoured Zn/Ni-MOF-2 nanosheets. Xu and co-workers constructed an iron-nickel-based single-crystal open capsular-MOF through a crystal-structure transformation (Fig. 6C).^94^ Nonhollow crystals of FeNi-MIL-88B were dispersed in a hot DMF solution of 2,4,6-tris(4-pyridyl)pyridine (tpy), affording uniform capsular-MOF nanocrystals. A detailed study of the growth mechanism revealed that the parent FeNi-MIL-88B with plenty of concave defects dissolved and released Fe^3+^, Ni^2+^, and BDC-NH_2_^2−^ under solvothermal conditions, followed by the construction of FeNi-MIL-88B-tpy on the surface of FeNi-MIL-88B and final formation of capsular FeNi-MIL-88B-tpy with openings in the nanocrystal walls. FeNi-MIL-88B-tpy with additional size-matching ligands (tpy) in the structure showed enhanced structural stabilities compared to FeNi-MIL-88B, thus driving the dissolution–regrowth process in hot DMF.

### 2.2 Core–shell bimetallic MOFs

In core–shell bimetallic MOFs, the shell and core MOFs are formed from different metal centres. Three well-established strategies have been exploited for fabricating core–shell bimetallic MOFs, namely, seed-induced growth, post-synthetic exchange, and one-pot synthesis.

#### 2.2.1 Seed-induced growth

**Fig. 7:**
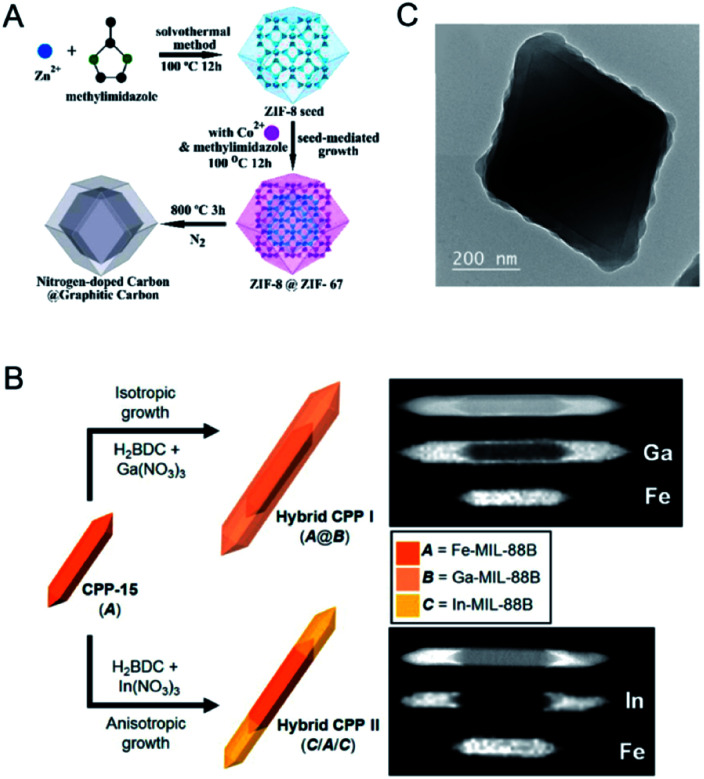
(A) Synthetic scheme for the preparation of core–shell ZIF-8@ZIF-67 crystals. Reproduced with permission from ref. 99. Copyright 2015, American Chemical Society. (B) Schematic representation of the preparation of two types of heterocompositional CPPs, Hybrid CPP I (A@B) and Hybrid CPP II (C/A/C), through the isotropic and anisotropic nanoscale growth of secondary CPs on CPP-15. Here, A = Fe-MIL-88B, B = Ga-MIL-88B, and C = In-MIL-88B. Reproduced with permission from ref. 102. Copyright 2013, American Chemical Society. (C) TEM image of UiO-66@ZIF-8. Reproduced with permission from ref. 103. Copyright 2015, Wiley-VCH.

Seed-induced growth has been demonstrated to be a powerful route for the synthesis of core–shell nanomaterials.^96,97^ Two MOFs with similar lattice parameters can be assembled to form core–shell MOFs by epitaxial growth. Kitagawa and co-workers demonstrated the first synthesis of core–shell bimetallic MOF single crystals by epitaxial growth.^98^ The hybrid material consisted of [Zn_2_(ndc)_2_-(dabco)]_*n*_ (**1**) as the core crystal and [Cu_2_(ndc)_2_-(dabco)]_*n*_ (**2**) as the shell crystal (ndc = 1,4-naphthalenedicarboxylate; dabco = diazabicyclo[2.2.2]octane) and was synthesized by immersing **1** in a solution of CuSO_4_·5H_2_O, H_2_ndc and dabco for the growth of **2**. Yamauchi and co-workers reported core–shell bimetallic ZIF materials with Zn-containing ZIF-8 as the core and Co-containing ZIF-67 as the shell (Fig. 7A).^99^ Tang and co-workers reported a sandwich structure with an inner core and an outer shell composed of MIL-101 with metal nodes of Fe^3+^, Cr^3+^ or both.^100^ Rosi and co-workers reported a domain building block (DBB) approach to construct a rich library of UiO-67 stratified MOF (sMOF) particles consisting of multiple concentric DBBs with core–shell and multilayered structures, such as UiO-67(Zr)⊂UiO-67(Hf), UiO-67(Hf)⊂UiO-67(Zr), and UiO-67(Zr)⊂UiO-67(Hf)⊂UiO-67(Zr).^101^

Oh and co-workers further demonstrated that lattice parameter variation of isostructural MOFs could lead to different growth behaviors in an isotropic or anisotropic fashion (Fig. 7B).^102^ They employed Fe-MIL-88B as the seed to direct the formation of Ga-MIL-88B and In-MIL-88B with analogous structures but different sizes of metal ions. Ga-MIL-88B isotropically grew on the entire surface of Fe-MIL-88B, while In-MIL-88B anisotropically grew in the *c*-direction at both tips of Fe-MIL-88B. The different behaviors are due to the size variation of the metal ions. The ion size of Ga(iii) is quite similar to that of Fe(iii), resulting in a good similarity in the cell parameters for growth in an isotropic manner. However, the ion size of In(iii) is larger than that of Fe(iii), leading to a significant increase in the *c* cell parameters of In-MIL-88B and thus the anisotropic growth of In-MIL-88B in the *c*-direction.

The epitaxial growth method is usually limited to the synthesis of core–shell MOFs with similar crystallographic parameters. The assembly of two MOFs with different crystal structures would inevitably suffer from the separate growth of MOFs in solution. To address this issue, it has been demonstrated that the use of capping agents can promote a conformal and oriented overgrowth of one MOF on another MOF with different crystal structures.^103–105^ Tsung and co-workers overgrew uniform ZIF-8 shells on monodisperse UiO-66 microcrystal cores to form UiO-66@ZIF-8 with the assistance of cetyltrimethylammonium bromide (CTAB).^103^ The authors proposed that CTAB could adsorb small and uniform ZIF-8 nuclei under sonication and then orient the nuclei to grow into a conformal shell (Fig. 7C). Li, Kitagawa and co-workers developed an internal extended growth method (IEGM) for the synthesis of a MOF-on-MOF structure [NH_2_-UiO-66(Zr) on NH_2_-MIL-125(Ti)], in which the two MOFs have distinct morphologies and crystal structures.^106^ NH_2_-UiO-66(Zr) was exposed to NH_2_-MIL-125(Ti) precursors with polyvinylpyrrolidone (PVP) as the structure-directing agent through microwave heating, resulting in the interspersion of NH_2_-UiO-66(Zr) particles on the NH_2_-MIL-125(Ti) nanotablet. During the synthesis, PVP covered NH_2_-UiO-66(Zr) NPs to coordinate Ti^4+^, thus allowing the nucleation and growth of NH_2_-MIL-125(Ti) around NH_2_-UiO-66(Zr) NPs. Various MOF-on-MOF materials, such as MIL-101(Cr) on NH_2_-MIL-125(Ti), MOF-76 (Tb) on NH_2_-MIL-125, could be successfully fabricated by this method.

Although capping agents can facilitate the integration of core MOFs and shell MOFs with distinct crystallographic parameters, the capping agent can potentially make the MOF–MOF interface complex and ill defined. In this regard, Zhou and co-workers reported the fabrication of MOF-on-MOF structures with mismatched lattices through retrosynthetic design without the use of capping agents.^107^ The authors chose stable PCN-222(Zr) (also known as MOF-545) as the core MOF, which was pretreated with linkers (H_2_BDC) of the shell MOF (MOF-5), resulting in the coordination of H_2_BDC on the surface of PCN-222(Zr). Further adding metal precursors (Zn(NO_3_)_2_) allowed the gradual formation of MOF-5 on the surface of PCN-222(Zr). This retrosynthetic method could be successfully applied to the synthesis of other MOF-on-MOF materials with stable MOFs (UiO-66 (Zr), UiO-67 (Zr), PCN-160 (Zr), MOF-808 (Zr), PCN-250 (Fe), and MIL-125(Ti)) as core MOFs and M(II) based carboxylate MOFs as the shell MOFs [MOF-177 (Zn), HKUST-1 (Cu), and MOF-1114 (Yb)] due to their mild synthetic conditions and relatively large crystal sizes.

#### 2.2.2 Post-synthetic exchange

**Fig. 8:**
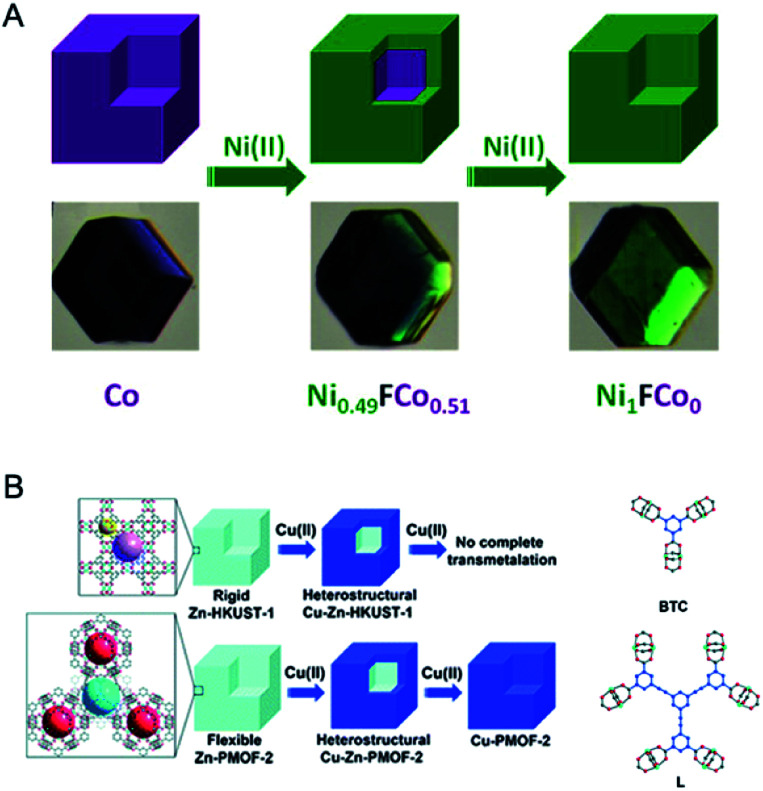
(A) Synthetic scheme for the preparation of core–shell heterostructures *via* selective transmetalations. Reproduced with permission from ref. 78. Copyright 2012, American Chemical Society. (B) Transmetalations in two MOFs (Zn-HKUST-1 and Zn-PMOF-2) with different framework flexibilities. Reproduced with permission from ref. 108. Copyright 2012, Royal Society of Chemistry.

Post-synthetic selective exchange of metal ions in the framework can be used to synthesize core–shell bimetallic MOFs. In the MOF crystal, metal sites in the core and close to the surface show different flexibilities and thus distinct reactivities. Therefore, through carefully controlling the post-synthetic metal exchange process, core–shell bimetallic MOFs can be obtained through selective transmetalation. Lah and co-workers reported a kinetically controlled replacement of the metal in the external region of the crystal to obtain core–shell bimetallic MOFs (Fig. 8A).^78^ A family of isostructural MOFs, M_6_(BTB)_4_(BP)_3_ (where M = Zn(ii) (1), Co(ii) (2), Cu(ii) (3), and Ni(ii) (4), BTB = 1,3,5-benzenetribenzoate, and BP = 4,4′-dipyridyl), were selected for the transmetalation reactions. Thermodynamically less stable 1 and 2 could undergo transmetalation to more stable 3 and 4, while the reverse transmetalation could not occur. The authors found that the metal sites in the external shell region of the crystal showed higher reactivities, thus reacting faster than those in the more rigid internal core. Therefore, by simply controlling the soaking time, core–shell heterostructures were formed through selective transmetalation.

In the subsequent work, Lah and co-workers further demonstrated the concept of selective transmetalation using two Zn-based MOFs with different ligand lengths, Zn-HKUST-1 and Zn-PMOF-2 (Zn_24_L_8_(H_2_O)_12_, L = 1,3,5-tris(3,5-dicarboxylphenylethynyl)benzene).^108^ In the methanol solution of Cu(ii), the transmetalation of Zn-HKUST-1 was incomplete even after three months. Zn-PMOF-2 showed a higher rate and extent in the transmetalation process due to its more flexible structure compared with Zn-HKUST-1 (Fig. 8B). For both MOFs, the exchange of the metal ions occurred selectively at the external shell, leading to a core–shell heterostructure.

#### 2.2.3 One-pot synthesis

One-pot synthesis can simplify the synthetic process and reduce steps for the separation and purification of intermediates, which is desirable but challenging.^109–111^ Core–shell bimetallic MOFs can be prepared in one pot, through mixing all the precursors of core and shell MOFs in the synthesis solution. It is of vital importance to control the nucleation and growth kinetics of the two MOFs in the synthesis solution, allowing the shell MOF to grow exclusively on the surface of the core MOF without self-nucleation. The control of the experimental parameters (*e.g.*, precursors, solvents, temperature, time, and modulators) is necessary to balance the rates of self-nucleation and growth of the core and shell MOFs, allowing them to assemble into a single nanostructure.

**Fig. 9:**
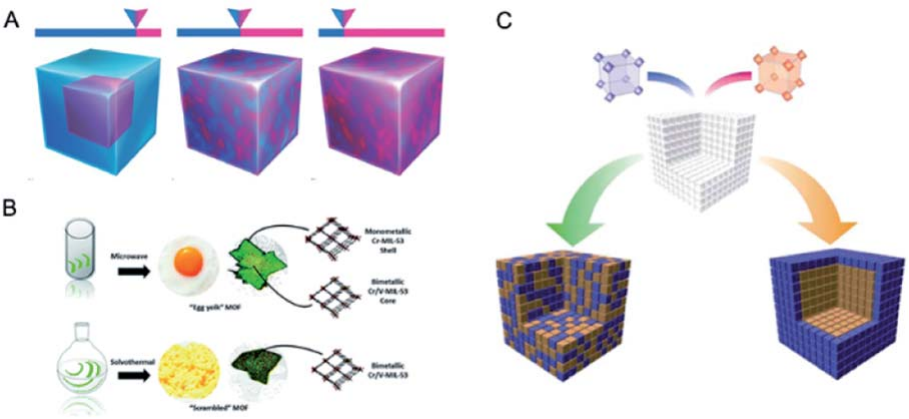
(A) Schematic illustration of Co_20_Zn_80_-ZIF, Co_50_Zn_50_-ZIF, and Co_80_Zn_20_-ZIF. Reproduced with permission from ref. 112. Copyright 2017, Wiley-VCH. (B) A one pot microwave procedure for creating mixed-metal “egg yolk” MOFs with a core of (Cr/V)-MIL-53 and a shell of Cr-MIL-53 (top). A solvothermal method for producing homogeneous mixed-metal MOFs (bottom). Reproduced with permission from ref. 113. Copyright 2017, Royal Society of Chemistry. (C) Schematic illustration of the construction of PCP crystals from binary crystal domain modules. Reproduced with permission from ref. 114. Copyright 2012, American Chemical Society.

Zou and co-workers investigated the reaction kinetics of Co^2+^ and Zn^2+^ with 2-MeIM for bimetallic Co/Zn ZIFs (Fig. 9A).^112^ The authors found that a low Co/Zn ratio resulted in Co-rich cores and Zn-rich shells. The growth went through a nucleation-growth separated process; Co^2+^ with higher reaction kinetics formed the cores first, followed by the growth of zinc shells. At a high Co/Zn ratio, the nucleation and growth processes were not separated, leading to a quick formation of solid solution Co/Zn ZIFs. Moreover, when adding Co^2+^ first, ZIF-67@ZIF-8/67 core–shell nanocrystals were obtained with tuneable core/shell thickness ratios. However, exchanging the sequence by adding Zn^2+^ first only formed agglomerates with irregular shapes.

Van Der Voort and co-workers reported egg-yolk bimetallic MOFs with a core of (Cr/V)-MIL-53 and a shell of Cr-MIL-53 using a one-pot microwave (MW) procedure.^113^ V^3+^ and Cr^3+^ salts were mixed with H_2_BDC in demineralized water in a MW for 3 h, resulting in the formation of (Cr/V)-MIL-53@ Cr-MIL-53 (Fig. 9B). For comparison, using a solvothermal method for 96 h resulted in one homogeneous phase of (Cr/V)-MIL-53 with uniform dispersion of V^3+^ and Cr^3+^. This work demonstrated that the synthesis time and method had a crucial influence on the metal dispersion in the crystals.

Kitagawa and co-workers controlled the kinetics of the crystal formation process for the preparation of core–shell MOFs in one pot by using precursors of distinct chemical reactivities (Fig. 9C).^114^ The authors employed [M(NO_2_-ip) (bpy)]0.5DMF·0.5MeOH (MCID-5⊃G, M = Zn, Mn, and Ni, NO_2_-ip = 5-nitroisophthalate, and bpy = 4,4′-bipyridyl) for the study. When using 5-nitroisophthalic acid (H_2_NO_2_-ip) as a precursor, the crystal formation rate of ZnCID-5⊃G was much quicker than that of MnCID-5⊃G. The one-pot reaction of H_2_NO_2_-ip and bpy with zinc and manganese perchlorate salts was conducted in a DMF/MeOH solution at 70 °C for 3 days, yielding crystals with a Zn-rich inner core and a Mn-rich outer shell. During the synthesis of Zn/MnCID-5⊃G, the nucleation process was dominated by the Zn rich crystals, which could facilitate the heterogeneous nucleation and growth of Mn-rich crystals. Moreover, additionally adding the precursors of NiCID-5⊃G with a moderate formation rate could yield Zn/Ni/MnCID-5⊃G crystals with a sandwich structure.

## 3. Applications of bimetallic MOFs

The mixing of metals in the SBUs of a MOF structure is widely recognized as an effective way to achieving optimal catalytic, optical, and electronic properties. A number of composition–property relationships have been established for many different MOF families.

The incorporation of second metal ions in the unstable SBUs of MOFs may enhance the stability of MOFs. For example, MOFs constructed from zinc(ii) paddlewheel [Zn_2_(OCO)_4_] SBUs frequently with low stability can show enhanced stability when exchanging the Zn^II^ ion for other metal ions (such as Cu^2+^ and Ni^2+^).^64,115–117^ Mg-MOF-74 can gain improved water stability by the incorporation of Ni^2+^ or Co^2+^ ions that are less likely to hydrolyze than Mg^2+^.^118^

The flexibility of MOFs can be modulated by adopting different metal ions in the SBUs to meet the requirements of desired applications, such as storage, separation, and sensing.^119^ Yeung, Goodwin and co-workers studied the thermal expansion in Zn/Cd ZIF-8.^35^ The coefficient of thermal expansion showed a continuous and monotonic decrease with the increase of Cd content in Zn/Cd ZIF-8. These results demonstrated that Cd substitution in ZIF-8 made the framework structure increasingly flexible, due to a weaker bonding of Cd^2+^ with 2-MeIM compared with Zn^2+^. Serre and co-workers reported the tuning of the breathing behaviour of MIL-53 by cation mixing.^55^ MIL-53(Cr–Fe) adopted a hydrated narrow pore (np) form below 343 K, and then changed to a closed pore (cp) form at 343 K and a large pore (lp) form upon further heating above 463 K. This breathing behaviour is different from that of MIL-53(Cr) and MIL-53(Fe), which showed a direct np to lp transformation and a two-step np to cp transformation, respectively.

The electronic properties of MOFs can be tailored through metal node engineering.^120,121^ Mixing different metals in the SBUs of MOFs may provide MOFs with excellent electrical conductivity for a variety of applications, such as semiconductors, supercapacitors, thermoelectrics, and resistive sensing. Chen, Shustova and co-workers prepared bimetallic MOFs, Cu_3−*y*_M_*y*_(BTC)_2_ (M = Co, Zn), and studied how the substitution of a secondary metal would affect the electronic properties of MOFs.^122^ Monometallic M-MOFs (M = Cu and Zn) and bimetallic CuZn-MOFs exhibited zero intensity near the Fermi level which were characteristic of insulators, while CuCo-MOFs exhibited semiconductor behaviour. Microwave conductivity measurements showed that effective conductivities were 0.1 × 10^−4^ and 3.5 × 10^−4^ S cm^−1^ for Cu_3_(BTC)_2_ and Cu_2.4_Co_0.6_(BTC)_2_, respectively. Density functional theory (DFT) calculations revealed that the Co incorporation into the Cu-BTC matrix resulted in a decrease in the band gap and thus a higher conductivity.

Bimetallic MOFs show enhanced physical and chemical properties due to the synergistic effect of the two metals. The remarkable features of bimetallic MOFs make them suitable for applications in a variety of fields, including gas adsorption, catalysis, energy storage and conversion, and luminescence sensing.

### 3.1 Gas adsorption

Developing efficient porous materials for gas adsorption is fundamentally and industrially important. Bimetallic MOFs with large surface areas, adjustable pore sizes, and open metal sites are promising candidates as adsorbents for gas adsorption. Moreover, the adsorption strength of a specific adsorbate in bimetallic MOFs can be optimized through tuning the compositions of bimetallic MOFs. Many bimetallic MOFs have been designed as excellent adsorbents for gas adsorption.

#### 3.1.1 Hydrogen adsorption

The hydrogen (H_2_) uptake of bimetallic MOFs is highly related to the structure of MOFs to store H_2_ and the unsaturated metal sites to bind with H_2_.^59^

**Fig. 10:**
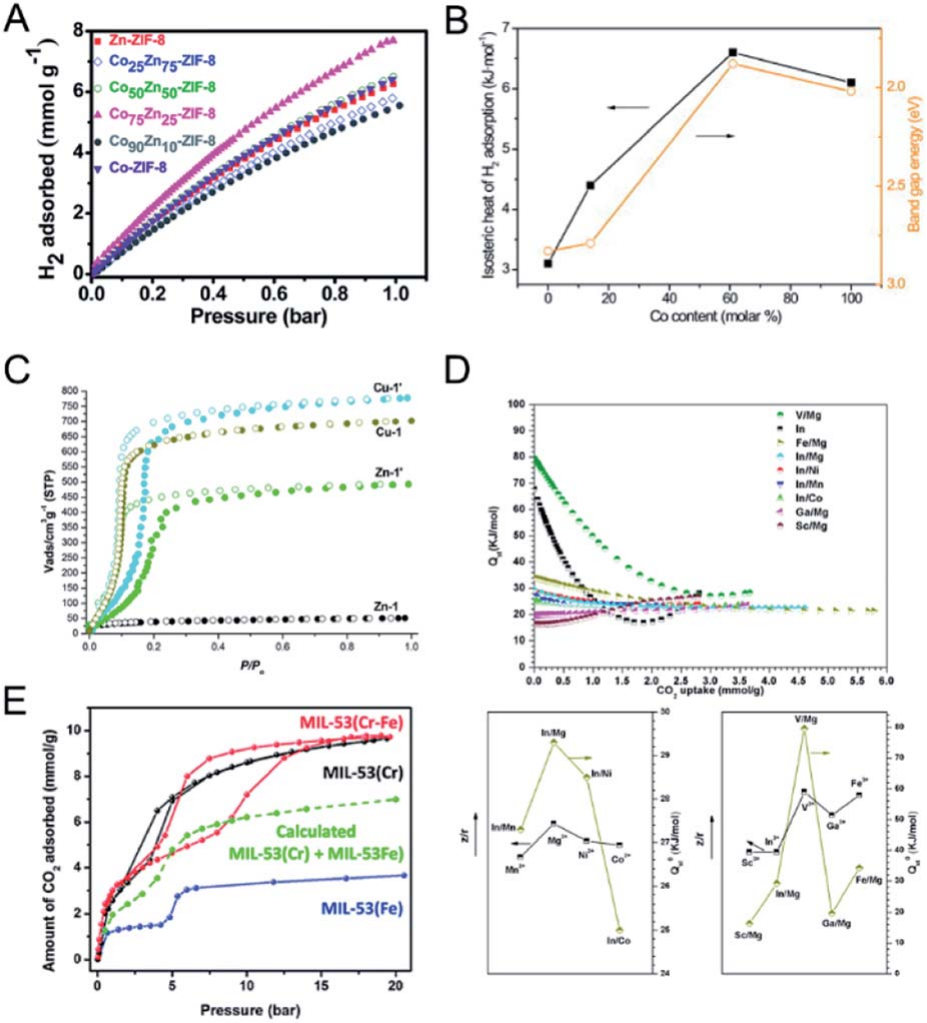
(A) H_2_ uptake isotherms of Zn-ZIF-8, Co-ZIF-8 and CoZn-ZIF-8 at 77 K. Reproduced with permission from ref. 123. Copyright 2016, Royal Society of Chemistry. (B) Hydrogen isosteric heat of adsorption, *Q*_st_ and band gap energy at 1.5% H_2_ coverage *versus* Co content (*x*) in Zn_1−*x*_Co_*x*_MOF-74. Reproduced with permission from ref. 51. Copyright 2011, Elsevier. (C) CO_2_ (195 K) adsorption isotherms of activated **Zn-1**, **Zn-1′**, **Cu-1**, and **Cu-1′**. Reproduced with permission from ref. 125. Copyright 2017, American Chemical Society. (D) Isosteric heat for CO_2_ for CPM-200s (top). Correlation between the isosteric heat at zero loading of CO_2_ (*Q*_st_^0^) and the charge-to-radius ratio (*z*/*r*) of metal ions for CPM-200 s (bottom). Reproduced with permission from ref. 126. Copyright 2016, American Chemical Society. (E) CO_2_ sorption isotherms at 283 K for the MIL-53 materials. Reproduced with permission from ref. 55. Copyright 2012, Royal Society of Chemistry.

Singh and co-workers prepared Co_*x*_Zn_100−*x*_-ZIF-8 with varying Co contents (*x* = 0, 25, 50, 75, 90 and 100).^123^ CoZn-ZIF-8 showed an enhancement of the surface area by ∼40% and the pore volume by ∼33% as compared to monometallic Zn-ZIF-8. At 77 K and 1 bar, Co_75_Zn_25_-ZIF-8 showed an enhancement of ∼23% in the H_2_ uptakes as compared to Zn-ZIF-8 (Fig. 10A). The enhanced H_2_ uptake capacities of CoZn-ZIF-8 could be attributed to the enhanced pore volume, surface area, microporosity, and heterogeneity in the pores.

Botas and co-workers prepared MOF-74 with different Co contents in the range of 0–100% for H_2_ adsorption at 0 °C and 10 bar.^51^ The H_2_ adsorption capacity of ZnCo-MOF-74 generally increased with the increase of Co content. The authors found a correlation between the isosteric heat of adsorption (*Q*_st_^0^) for H_2_ and the band gap energy values of Zn_1−*x*_Co_*x*_MOF-74 (Fig. 10B), implying that H_2_-metal cluster interactions have a more decisive role in H_2_ adsorption.

Long and co-workers exchanged Mn^2+^ ions in Mn_3_[(Mn_4_Cl)_3_(BTT)_8_(CH_3_OH)_10_]_2_ with selected cations (M = Li^+^, Cu^+^, Fe^2+^, Co^2+^, Ni^2+^, Cu^2+^, Zn^2+^) to tune the interaction strength between H_2_ and unsaturated metal centres in the frameworks.^62^ All the materials showed similar H_2_ uptakes ranging from 2.00 wt% to 2.29 wt% at 77 K and 900 torr. However, the materials showed significantly different uptake slopes at very low pressure, indicating different metal–hydrogen binding strengths in the materials. The results showed that Mn^2+^, Fe^2+^, and Co^2+^ ions yielded the strongest H_2_ binding among the cations studied.

#### 3.1.2 Carbon dioxide adsorption

Developing adsorbents with high physicochemical stabilities, high carbon dioxide (CO_2_) capacity and selectivity, and low regeneration costs is still a challenge. MOFs are promising porous crystalline materials with high porosities, surface areas and open metal sites for CO_2_ capture.^124^

The incorporation of a second metal in MOFs can improve the stability of MOFs and provide stronger interaction with CO_2_. Sun and co-workers used the metal ion exchange strategy to fabricate bimetallic MOFs with improved stability and CO_2_ adsorption capacity.^125^ Zn^II^ ions in **Zn-1** ([Zn_3_(L)_2_(dabco)(H_2_O)]·9DMF, H_3_L = [1,1′:3′,1′′-terphenyl]-4,4′′,5′-tricarboxylic acid) were partially replaced by Cu^II^ ions to fabricate bimetallic **Zn-1′**. The copper(ii) analogue, **Cu-1**, was exchanged with Zn^II^ ions to fabricate **Cu-1′**. **Zn-1′** showed an enhanced N_2_ adsorption amount (2063 m^2^ g^−1^) than **Zn-1** (59 m^2^ g^−1^), due to the high stability of the hybrid framework. **Cu-1** and **Cu-1′** showed similar N_2_ adsorption amounts. At 195 K and 1 bar, the CO_2_ uptakes of **Zn-1**, **Zn-1′**, **Cu-1**, and **Cu-1′** were 52, 490, 703, and 770 m^3^ g^−1^, respectively. Both bimetallic **Zn-1′** and **Cu-1′** exhibited improved adsorption capacities than their pristine counterparts. Moreover, bimetallic **Zn-1′** and **Cu-1′** showed large hysteresis in the CO_2_ adsorption (Fig. 10C), implying enhanced interactions between the frameworks and CO_2_ molecules. Xiao, Li and co-workers doped Mg in MIL-101(Cr) to synthesize bimetallic MIL-101(Cr, Mg), showing improved CO_2_ adsorption capacity.^54^ The CO_2_ adsorption capacity of MIL-101(Cr, Mg) reached 3.28 mmol g^−1^ at 298 K and 1 bar, with an increase of 40% in comparison with MIL-101(Cr). The enhanced CO_2_ adsorption of MIL-101(Cr, Mg) was attributed to the higher surface area and stronger adsorptive sites for CO_2_ due to the doping of Mg.

Bu, Feng and co-workers reported the cooperative effect of dissimilar metals in the SBUs of MOFs for CO_2_ adsorption.^126^ The authors prepared a family of isostructural heterometallic MOFs (M^II^_2_M^III^(μ_3_-OH)(CO_2_)_6_, CPM-200 series, CPM = crystalline porous materials) with combinations of trivalent (In^3+^, Ga^3+^, Fe^3+^, V^3+^, and Sc^3+^) and divalent metals (Mg^2+^, Mn^2+^, Co^2+^, and Ni^2+^) in the SBUs.^126^ At 273 K and 1 bar, the CO_2_ uptakes of the CPM-200 series, in cm^3^ g^−1^, were in the order of –Fe/Mg (207.6) > –In/Mg (190.8) > –V/Mg (155.4) > –In/Co (136.9) > –Ga/Mg (136.2) > –In/Mn (126.5) > –Sc/Mg (122.4) > –In/Ni (100.4). Among the CPM-200 series, CPM-200-Sc/Mg showed the highest *Q*_st_^0^ for CO_2_ (−79.6 kJ mol^−1^). The authors demonstrated a strong correlation between the charge-to-radius ratio (*z*/*r*) of metal ions in MOFs and *Q*_st_^0^ for CO_2_ (Fig. 10D).

In addition to the open metal sites, the pore architecture is of vital importance for CO_2_ capture performance. Hill and co-workers reported the exchange of Zr in UiO-66 for Ti to yield bimetallic UiO-66 with a smaller pore size and higher adsorption enthalpy for an enhanced CO_2_ uptake.^80^ With the increase of Ti substitution, the octahedral cages shrank by ∼1 Å and became increasingly broader. The CO_2_ uptake at 273 K of UiO-66(Zr_100_), UiO-66(Ti_32_) and UiO-66(Ti_56_) was 2.2, 2.3 and 4 mmol g^−1^, respectively. Considering that theoretical enhancement in the CO_2_ gravimetric uptake of UiO-66(Ti_100_) is ∼19% while UiO-66(Ti_56_) showed an enhancement of 81% compared with UiO-66(Zr_100_), the authors proposed that both the decrease of pore sizes and stronger adsorption characteristics of Ti(iv) contributed to the enhanced CO_2_ uptake in Ti-exchanged UiO-66.

Moreover, the structural flexibility change of bimetallic MOFs will lead to a different CO_2_ sorption behaviour compared with their single cation analogues. Serre and co-workers reported different CO_2_ sorption isotherms of bimetallic MIL-53(Cr–Fe) compared to monometallic MOFs (Fig. 10E).^55^ CO_2_ sorption tests at 283 K showed that the np to lp transition of MIL-53(Cr–Fe) occurred at a pressure of ∼10 bar, which was an intermediate between those of MIL-53(Cr) (3 bar) and MIL-53(Fe) (20 bar). This result demonstrated that MIL-53(Cr–Fe) was an intermediate between MIL-53(Cr) (‘easy’ to open) and MIL-53(Fe) (‘hard’ to open) in terms of ease of pore opening.

### 3.2 Catalysis

#### 3.2.1 Organocatalysis

The use of the inorganic nodes of MOFs as catalytically active sites has several advantages, including a good dispersion of the inorganic nodes within the framework to well expose the active sites and a well-defined structure of the inorganic nodes for precise structural characterization and computational modeling.^127,128^ When using monometallic MOFs as catalysts, the metal ions in the inorganic nodes need to undergo a change of the coordination environment, which may sometimes lead to the collapse of the frameworks. It is a good idea to design a bimetallic MOF, in which one metal contributes to structural stability and the other metal acts as an active centre for catalysis. This design can also allow the isolation and stabilization of active metal sites to achieve high activity, selectivity and stability.^129–135^ For example, Chen and co-workers prepared (Cu_*x*_Ru_1−*x*_)_3_-(BTC)_2_ (CuRhBTC) for propylene hydrogenation.^136^ CuRh(33%)BTC exhibited an activity of 1600 μmol g_catalyst_^−1^ min^−1^ for propane production. The CuBTC MOF showed no activity under the reaction conditions, and RhBTC was unstable upon exposure to air. The inactive Cu ions in CuRhBTC played an important role in stabilizing the MOF framework and preventing the reduction of the incorporated Rh^2+^.

**Fig. 11:**
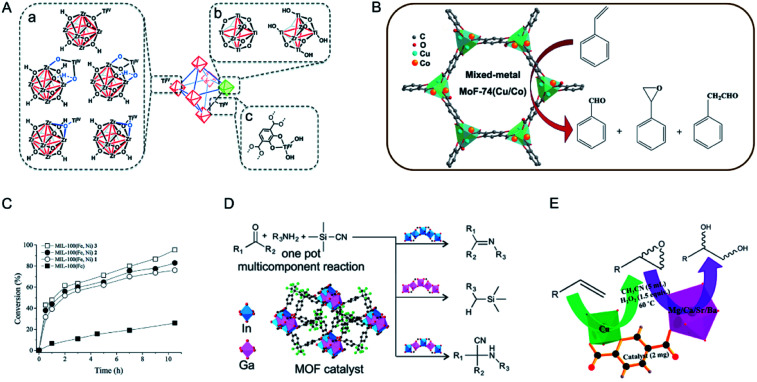
(A) Proposed structures for Ti^IV^ ions supported on the nodes (a), as the nodes (b), and on the struts (c) of UiO-66. Reproduced with permission from ref. 137. Copyright 2015, Royal Society of Chemistry. (B) MOF-74(Cu/Co) with different Cu/Co ratios as catalysts in the oxidation of styrene with O_2_ under solvent-free and mild reaction conditions. Reproduced with permission from ref. 139. Copyright 2016, Elsevier. (C) Time conversion plot of β-pinene, using as a catalyst either the mixed-metal MIL-100(Fe, Ni) **1–3** or homometallic MIL-100(Fe). Reproduced with permission from ref. 144. Copyright 2019, Royal Society of Chemistry. (D) Different behaviours of GaPF-1, InPF-11β and In_*x*_Ga_1−*x*_MOF as heterogeneous catalysts in the three-component, one pot Strecker reaction. Reproduced with permission from ref. 149. Copyright 2015, American Chemical Society. (E) Bimetallic MOFs with two different metals catalyzing two kinds of reactions, *viz.*, olefin to its epoxide followed by epoxide ring opening to afford the corresponding vicinal diol in a sequential manner. Reproduced with permission from ref. 150. Copyright 2016, American Chemical Society.

Although catalytically active metal ions can be introduced through immobilization on the SBUs or organic linkers, the incorporation of a second metal on the SBUs of MOFs for bimetallic MOFs results in distinct catalytic properties compared with other ways. In this regard, Farha, Hupp, Nguyen and co-workers compared the catalytic activity of UiO-66(Zr) functionalized with Ti^IV^ ions as part of the node (Ti-UiO-66), attached to the node (UiO-66-Ti_ex_), and on the organic linker (UiO-66-Cat-Ti) in the oxidation of cyclohexene.^137^ These three materials had Ti^IV^ ions with different coordination environments at different sites of the UiO-66 support (Fig. 11A). Three UiO-66-based catalysts showed the catalytic activity in the order of Ti-UiO-66 ≫ UiO-66-Cat-Ti > UiO-66-Ti_ex_, indicating that tetrahedral Ti^IV^ ions were more active than the more saturated octahedral Ti^IV^ ions.

Monometallic MOF catalysts usually exhibit either unsatisfactory activity or selectivity. Bimetallic MOFs can show a synergistic effect between the two metal ions to activate the reactants and reduce the reaction energy barrier in heterogeneous catalysis.^31^ Bimetallic MOFs have shown promising catalytic performances in a variety of reactions, including oxidation reactions,^56,76,138–141^ reduction reactions,^142,143^ condensation reactions,^144–146^ addition reactions,^53,147^ and substitution reactions.^148^

Zhu, Fan and co-workers applied bimetallic MOF-74(Cu/Co) with different Cu/Co ratios in styrene oxidation with O_2_ (Fig. 11B).^139^ MOF-74(Cu) showed a low catalytic activity (0.6% conversion) but an absolute selectivity for benzaldehyde. MOF-74(Co) possessed a high catalytic activity (47.3% conversion) but a low selectivity (35%) for benzaldehyde. The incorporation of Co^2+^ in MOF-74 (Cu) could significantly enhance the conversion of styrene. With the increase of Co^2+^ content in MOF-74(Cu/Co), the conversion of styrene increased while the selectivity for benzaldehyde decreased. A physical mixture of MOF-74(Cu) and MOF-74(Co) exhibited a lower activity than MOF-74(Cu/Co), indicating the synergistic effect of Cu^2+^ and Co^2+^ in the same framework.

Garcia, Serre and co-workers synthesized a series of MIL-100(Fe, Ni) materials with different metal ratios as catalysts for the Prins reaction.^144^ MIL-100(Fe, Ni) (**1–3**) samples contained a Ni amount of 1%, 3%, and 5%, respectively. In the Prins reaction between β-pinene and paraformaldehyde, monometallic MIL-100(Fe) only showed a low conversion (<20%). Bimetallic MIL-100(Fe, Ni) afforded higher conversions between 70 and 100%. MIL-100(Fe, Ni) **3** with the highest Ni percentage showed the highest activity, with a total conversion to nopol in 10 h (Fig. 11C). The authors proposed that the incorporation of Ni^II^ could cause the distortion of the framework to facilitate the access to the Fe^III^ sites, thus enhancing the catalytic activity of the catalyst.

Park and co-workers synthesized a solid solution Zn–Co ZIF (CZ-ZIF) for chemical fixation of CO_2_.^147^ In the cycloaddition reaction of CO_2_ with epichlorohydrin (ECH), ZIF-8 showed an excellent ECH conversion of 98% but a low selectivity of 33% towards epichlorohydrin carbonate (ECC). ZIF-67 exhibited high selectivity to ECC (98%) but an inferior conversion of 66%. Solid solution CZ-ZIF afforded both high conversion of ECH (94%) and high selectivity (98%) to the desired ECC. The bimetallic CZ-ZIF could combine the advantageous properties of Zn (high activity) and Co (high selectivity and low leaching), achieving enhanced catalytic performance compared to its monometallic counterparts.

Multicomponent reactions and cascade reactions which require the cooperation of different active sites can be accomplished in bimetallic MOFs.^149–152^ Monge and co-workers demonstrated the control of the catalytic activity and selectivity of bimetallic MOFs in a multicomponent one pot Strecker reaction by modulating specifically selected metal ratios (Fig. 11D).^149^ In the A^3^ reaction between benzaldehyde, trimethylsilyl cyanide (TMSCN), and aniline, monometallic GaPF-1 [Ga(OH)(hfipbb)] and InPF-11β [In(O_2_C_2_H_4_)_0.5_(hfipbb)] (H_2_hfipbb = 4,4′-(hexafluoroisopropylidene) bis(benzoic acid)) gave only aldehyde cyanosilylation and imine as the main product, respectively. Solid-solution bimetallic MOFs [In_*x*_Ga_1−*x*_(O_2_C_4_H_4_)_0.5_(hfipbb), *x* = 0.72, 0.55, and 0.28] could selectively yield the final Strecker product (α-aminonitrile) with different reaction rates. InGaPF-3 with the least amount of In showed the highest reaction rate, with 96% conversion in 0.33 h. Koner and co-workers reported a series of bimetallic MOFs based on copper and alkaline-earth metals (Mg, Ca, Sr, and Ba) to effectively catalyse olefin epoxidation and subsequent epoxide ring opening (Fig. 11E).^150^ In the tandem reaction, Cu^II^ acted as an active centre in epoxidation reactions and alkaline-earth metal ions served as Lewis acidic sites for epoxide ring opening subsequently. These MOFs with different alkaline-earth metals showed different reaction rates, increasing in the order of Mg < Ca < Sr ≈ Ba. The authors proposed that the increase of the size of the alkaline-earth metals led to a greater number of ligands accommodated in the coordination sphere and thus formed more open sites to enhance the catalytic activity.

#### 3.2.2 Photocatalysis

Bimetallic MOFs can also act as excellent catalysts for photocatalytic reactions, such as organic reactions, water splitting, CO_2_ reduction, and organic pollutant degradation.

**Fig. 12:**
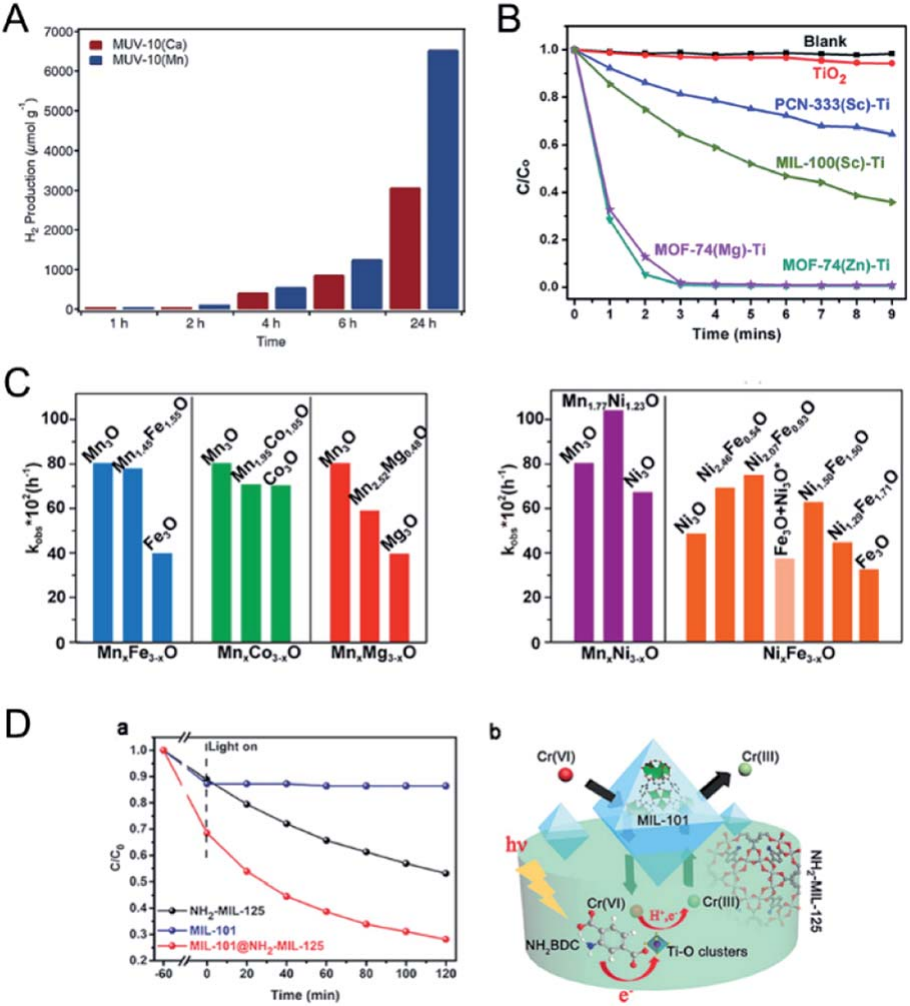
(A) Photocatalytic generation of hydrogen after 24 hours of irradiation using MUV-10(Ca) and MUV-10(Mg). Reproduced with permission from ref. 153. Copyright 2018, Wiley-VCH. (B) Photodegradation of MB using no catalyst (blank), TiO_2_, PCN-333(Sc)–Ti, MIL-100(Sc)–Ti, MOF-74(Zn)–Ti and MOF-74(Mg)–Ti with 300 W xenon light irradiation. Reproduced with permission from ref. 158. Copyright 2016, Royal Society of Chemistry. (C) Comparison between *k*_obs_ of MTV-MOFs in a domain metal arrangement (left) and well-mixed metal arrangement (right) and those of their corresponding single component MOFs. Reproduced with permission from ref. 40. Copyright 2016, American Chemical Society. (D) Adsorption and photocatalytic degradation toward Cr^VI^ with MIL-101, NH_2_-MIL-125, and MIL-101@NH_2_-MIL-125 (a). Illustration of the enhanced photocatalytic degradation process towards Cr^VI^ with micro/mesoporous MIL-101@NH_2_-MIL-125 materials (b). Reproduced with permission from ref. 106. Copyright 2017, Wiley-VCH.

Ti-based MOFs have shown great promise in photocatalysis. The incorporation of second metal ions in the SBUs of Ti-based MOFs can greatly enhance the photocatalytic activity. Martí-Gastaldo and co-workers reported two Ti-based MOFs (MUV-10, Ti^IV^_3_M^II^_3_(μ_3_-O)_2_(btc)_4_-(H_2_O)_6_, M = Ca^II^, Mg^II^) by metal doping for photocatalytic H_2_ production (Fig. 12A).^153^ The authors found that heterometallic clusters served as excellent platforms to manipulate the electronic structure and in turn regulate the photoactivity. MUV-10(Mn) could produce 6500 μmol g^−1^ of H_2_ after 24 hours under the irradiation of a Xe lamp (300 W), showing two times higher activity than that of MUV-10(Ca). However, few Ti-based MOFs have been reported due to the high reactivity of Ti precursors. Alternatively, Ti can be incorporated into MOFs through post-synthetic exchange methods. For example, Ti-substituted UiO-66 (Zr/Ti) materials have shown enhanced photocatalytic performance compared to monometallic UiO-66(Zr).^154–157^ Zhou and co-workers synthesized four Ti based-MOFs, PCN-333(Sc)–Ti, MIL-100(Sc)–Ti, MOF-74(Zn)–Ti and MOF-74(Mg)–Ti, using a high valence metathesis and oxidation (HVMO) method for photodegradation of methylene blue (MB).^158^ The metal exchange ratios of PCN-333(Sc), MIL-100(Sc), MOF-74(Zn) and MOF-74(Mg) were 85.9%, 52.0%, 100%, and 35.1%, respectively (Fig. 12B). PCN-333(Sc)–Ti and MIL-100(Sc)–Ti showed 35% and 64% degradation of MB under a 300 W Xe lamp for nine minutes, respectively. MOF-74(Zn)–Ti and MOF-74(Mg)–Ti both showed conversions up to 98% in three minutes. All the synthesized Ti based-MOFs showed enhanced activities compared to TiO_2_ and monometallic MOFs.

Deng and co-workers demonstrated the synergistic effect of two different metals within the SBUs of MOFs for the photo-oxidation of 1,5-dihydroxynaphthalene (DHN).^40^ The authors synthesized a series of multivariate MOFs (MTV-MOFs) with a formula of (M_3_O)_2_(TCPP-M)_3_ (TCPP = tetrakis (4-carboxyphenyl) porphyrin). MTV-MOFs composed of Mn_*x*_Ni_3−*x*_O and Ni_*x*_Fe_3−*x*_O SBUs showed a well-mixed spatial arrangement of metals, which exhibited enhanced conversion rates compared to their corresponding single component MOFs. Detailed characterization demonstrated that band gaps changed dramatically when different metals were present in the same SBU with a synergistic effect, leading to effective electron/hole transfer to oxygen for a high reaction rate. For comparison, in the case of MTV-MOFs composed of Mn_*x*_Fe_3−*x*_O, Mn_*x*_Co_3−*x*_O and Mn_*x*_Mg_3−*x*_O SBUs, the two kinds of metals were in different SBUs and thus showed negligible interactions due to the separation by organic linkers (Fig. 12C). Therefore, these bimetallic MOFs exhibited the highest catalytic rates among their corresponding single component MOFs.

Core–shell MOFs have been successfully applied in photocatalysis, in which the shell MOF can accumulate/recognize reactants and the core MOF with catalytically active sites can transform the reactants to products.^106,107,159^ Kitagawa and co-workers applied MIL-101(Cr)@NH_2_-MIL-125(Ti) in the adsorption-photocatalytic removal of Cr^VI^ (Fig. 12D).^106^ MIL-101(Cr)@NH_2_-MIL-125(Ti) showed enhanced adsorption capacity on Cr^VI^ (3.16 mg g^−1^) compared to those of NH_2_-MIL-125(Ti) (1.12 mg g^−1^) and MIL-101(Cr) (1.28 mg g^−1^). MIL-101(Cr)@NH_2_-MIL-125(Ti) removed 72% of Cr^VI^ from the solution under visible light irradiation. However, MIL-101(Cr) showed no activity and NH_2_-MIL-125(Ti) removed only 47% of Cr^VI^ under similar conditions. The MOF on MOF heteroarchitecture could combine the high adsorption capacity of MIL-101(Cr) and photocatalytic activity of NH_2_-MIL-125(Ti) to exhibit high efficiency in photocatalysis.

#### 3.2.3 Electrocatalysis

Bimetallic MOFs have been designed for electrocatalysis, such as the oxygen reduction reaction (ORR),^160^ oxygen evolution reaction (OER),^134,161–166^ hydrogen evolution reaction (HER),^167,168^ and electrochemical CO_2_ reduction.^169,170^

**Fig. 13:**
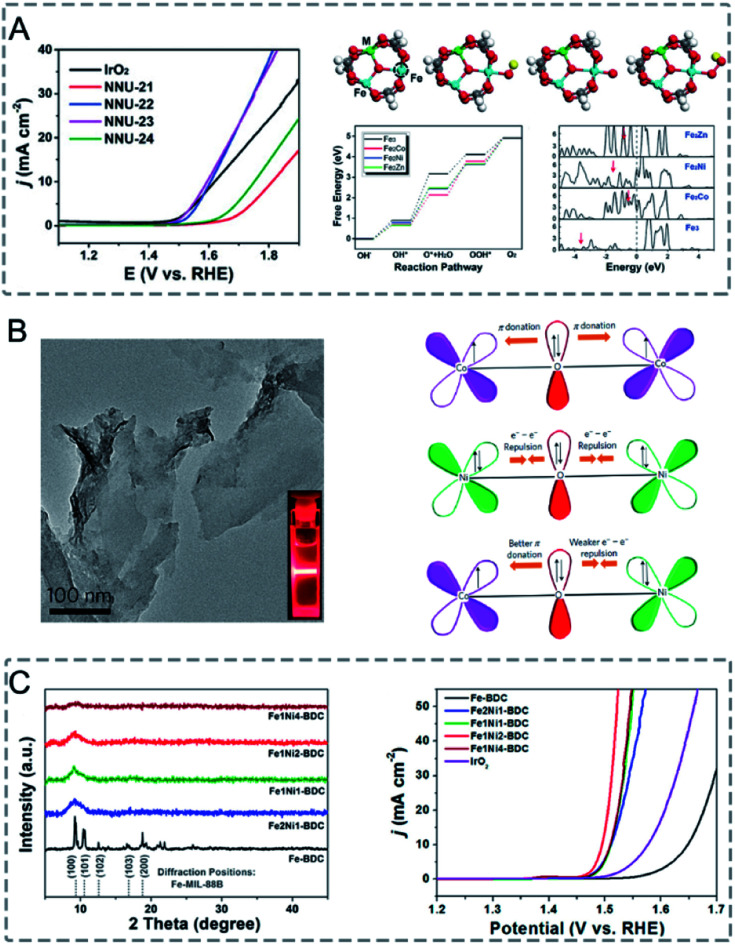
(A) LSV curves of IrO_2_ and NNU-21–24 for the OER in 0.1 m KOH (left). Initial structure of the Fe_2_M cluster, followed by the adsorption of OH*, O*, and OOH* intermediates on the Fe site. The active site is marked by a dashed circle. The free energy profile for the OER pathway and projected density of states of the Fe_2_M-cluster (right). Red arrows denote the d-band center. Reproduced with permission from ref. 163. Copyright 2018, Wiley-VCH. (B) TEM image of NiCo-UMOFNs (left). Schematic representation of the electronic coupling between Co and Ni in UMOFNs (right). Reproduced with permission from ref. 174. Copyright 2016, Nature Publishing Group. (C) XRD patterns of Fe-BDC, Fe_2_Ni_1_-BDC, Fe_1_Ni_1_-BDC, Fe_1_Ni_2_-BDC, and Fe_1_Ni_4_-BDC (diffraction positions of Fe-MIL-88B are shown in the bottom) (left). LSV curves of Fe-BDC, Fe_2_Ni_1_-BDC, Fe_1_Ni_1_-BDC, Fe_1_Ni_2_-BDC, Fe_1_Ni_4_-BDC, and IrO_2_ in 1 M KOH at a scan rate of 5 mV s^−1^ (right). Reproduced with permission from ref. 164. Copyright 2019, American Chemical Society.

In bimetallic MOFs, the electron configurations and d-band centres can be tailored through the mix of different metals in the SBUs, offering opportunities to enhance the electrocatalytic performance.^122,171^ Li, Lan and co-workers prepared four isostructural MOFs (NNU-21–24) based on Fe_2_M (M = Fe, Co, Ni, Zn) clusters bridged with biphenyl-3,4′,5-tricarboxylic acid (BPTC) ligands for the electrocatalysis of the OER.^163^ Among the four synthesized MOFs, monometallic NNU-21 (Fe_3_-BPTC) showed lower OER performance than bimetallic MOFs (NNU-22 (Fe_2_Co-BPTC), NNU-23 (Fe_2_Ni-BPTC) and NNU-24 (Fe_2_Zn-BPTC)) (Fig. 13A). NNU-23 exhibited the best OER performance with an overpotential of 365 mV at a current density of 10 mA cm^−2^ in 0.1 M KOH. Moreover, all the catalysts exhibited high stability with almost no decrease of activity after 2000 cycles. DFT calculation revealed that the incorporation of a second metal (Co, Ni or Zn) into the Fe cluster could induce the d-band centre to be close to the Fermi level, leading to a stronger binding interaction between the O* intermediate and catalysts, thus improving the OER performance. In addition to solid solution bimetallic MOFs, MOF@MOF structures can also show a synergistic effect in electrocatalysis.^172^ Zhu, Sun and co-workers grew Fe-MOF NPs onto Ni-MOF nanosheets (Ni-MOF@Fe-MOF) for the OER.^173^ The deposition of catalytically inert Fe-MOF on Ni-MOF could enhance the electrocatalytic performance. Ni-MOF@Fe-MOF exhibited an overpotential of 265 mV to reach a current density of 10 mA cm^−2^ in 1.0 M KOH, which was lower than that of Ni-MOF (370 mV). The enhanced performance of Ni-MOF@Fe-MOF could be attributed to the synergistic effect between Ni active centres and Fe species.

However, bimetallic MOFs usually suffer from poor conductivity, blockage of active metal centres by organic linkers, and low stability, which dramatically limit their utilization as electrocatalysts. To address these challenges, thinning bimetallic MOFs into nanosheets, fabricating an amorphous structure with plenty of defects, and integrating bimetallic MOFs with conductive supports have been proposed to enhance the electrocatalytic performance of bimetallic MOFs.

Thinning bimetallic MOFs into two-dimensional (2D) nanosheets can enhance mass transport and electron transfer and maximize unsaturated metal sites on the surfaces to improve the electrocatalytic performance.^8,160,174–177^ Liu, Zhao, Tang and co-workers reported ultrathin NiCo bimetallic MOF nanosheets (NiCo-UMOFNs) as effective electrocatalysts for the OER (Fig. 13B).^174^ NiCo-UMOFNs on a glassy-carbon electrode showed an overpotential of 250 mV at a current density of 10 mA cm^−2^ in 1.0 M KOH solution, smaller than those of Ni-UMOFNs (321 mV), Co-UMOFNs (371 mV), bulk NiCo-MOFs (317 mV) and commercial RuO_2_ (279 mV). XAFS analysis and DFT calculations demonstrated that the coordinatively unsaturated metal atoms were the dominating active centres and the synergistic effect between Co and Ni further improved the electrocatalytic OER activity. Electron transfer from the Ni^2+^ sites to the Co^2+^ sites could enhance the interaction between Ni^2+^ sites and water, thus offering a lower energy barrier for water oxidation. Oh and co-workers prepared bimetallic conductive 2D MOFs (Co_*x*_Ni_*y*_-CATs) with varied ratios of Co^2+^ to Ni^2+^ for the ORR in 0.1 M NaClO_4_ and 0.02 M PBS electrolytes.^160^ Ni-CAT showed a high onset potential of 0.47 V (*vs.* the reversible hydrogen electrode, RHE, the same below if not mentioned), but a low diffusion limiting current density at 0.0 V (−3.62 mA cm^−2^). However, Co-CAT displayed a high diffusion limiting current density (−5.59 mA cm^−2^) but a low onset potential (0.42 V). Bimetallic Co_0.27_Ni_0.73_-CAT combined the advantages of Co-CAT (effective adsorption for O_2_) and Ni-CAT (high conductivity), showing a diffusion-limiting current density (−5.68 mA cm^−2^) and an onset potential (0.46 V). With the increase of the proportion of Ni from 50% to 73%, the onset potential of Co_*x*_Ni_*y*_-CATs showed a slight increase. Further increasing the proportion of Ni to 86% resulted in a decrease in diffusion-limiting current density. Rotating ring disk electrode (RRDE) investigation showed that the electron transfer number of Co-CAT, Ni-CAT, and Co_0.27_Ni_0.73_-CAT was 3.91, 3.31, and 3.95, respectively. The bimetallic Co_0.27_Ni_0.73_-CAT was more preferable for a four-electron process.

In MOFs with good crystallinity, the metal sites are confined in the crystalline frameworks and blocked by the organic linkers, which is not good for electrocatalysis. In this regard, low-crystalline bimetallic MOFs with highly exposed active sites and porous structures hold great promise for electrocatalysis. Mai and co-workers reported the synthesis of low-crystalline bimetallic Fe/Ni-MOF NPs (Fe_*x*_Ni_*y*_-BDC) for the OER.^164^ The addition of Ni precursors in the synthesis solution of Fe-BDC (Fe-MIL-88B) caused mismatched coordination with organic linkers, thus resulting in the loss of the long-range ordered structure and formation of rich defects (Fig. 13C). Fe_1_Ni_2_-BDC showed a small overpotential of 260 mV at a current density of 10 mA cm^−2^ and a high Faradaic efficiency (FE) of 99.5% for the OER in 1.0 M KOH. The superior catalytic performance of Fe_1_Ni_2_-BDC could be attributed to the exposed active sites, synergistic effect between Fe and Ni, and fast charge transfer in the amorphous structure.

The integration of bimetallic MOFs with functional materials can enhance the characteristics of MOFs with improved electrical conductivity, chemical stability and mechanical strength for electrocatalytic applications.^6,167,178–180^ Lu and co-workers reported the *in situ* growth of amino-functionalized bimetallic NH_2_-MIL-88B(Fe_2_Ni) (NFN-MOF) on 3D microporous nickel foam (NF) as an efficient bifunctional electrocatalyst for overall water splitting.^181^ In a 1.0 M KOH electrolyte, the synthesized NFN-MOF/NF exhibited overpotentials of 240 and 87 mV at a current density of 10 mA cm^−2^ for the OER and HER, respectively. For the overall water splitting with NFN-MOF/NF as both the anode and cathode, a cell voltage of only 1.56 V was required to achieve a current density of 10 mA cm^−2^. The excellent catalytic performance of NFN-MOF/NF can be attributed to the synergistic effect between Ni and Fe metal ions in the MOF, and a positive coupling effect between the MOF and NF.

### 3.3 Energy storage and conversion

**Fig. 14:**
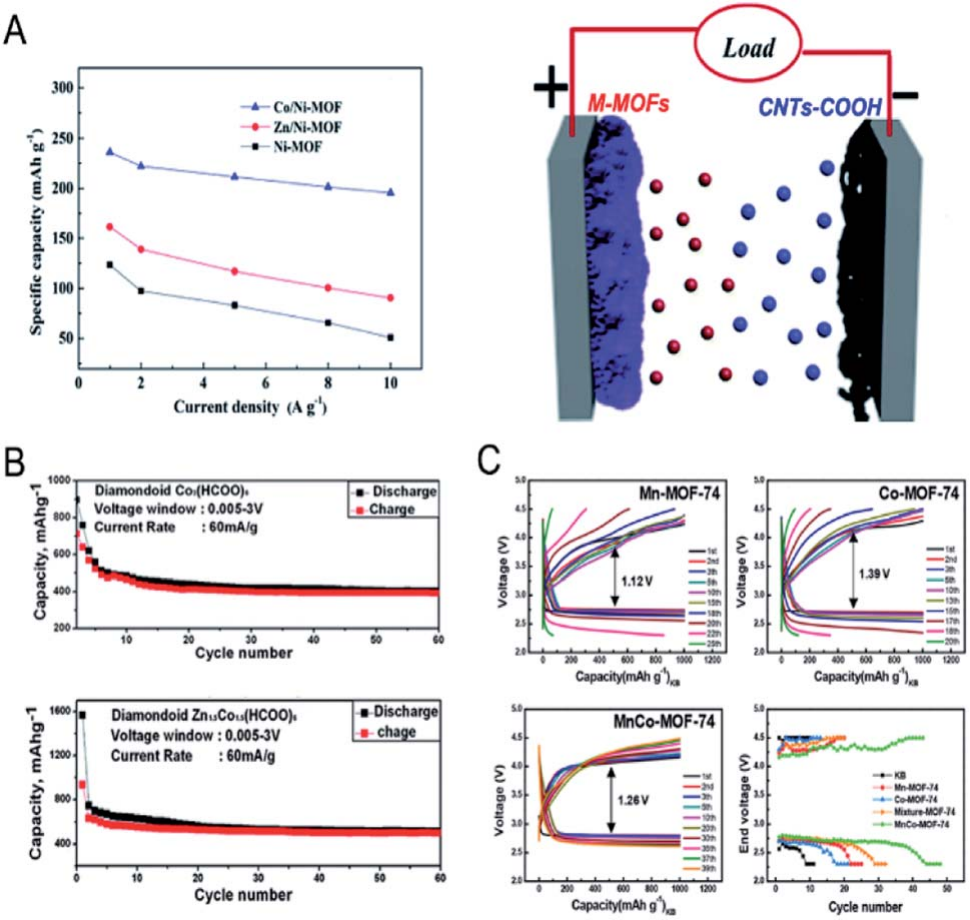
(A) Specific capacity of pristine Ni-MOF, Zn/Ni-MOF and Co/Ni-MOF at various current densities (left). An abstract illustration of hybrid supercapacitors containing M-MOFs and CNTs–COOHs as a positive and negative electrode, respectively (right). Reproduced with permission from ref. 190. Copyright 2017, Royal Society of Chemistry. (B) Capacity *vs.* cycle number plot for FOR3 (top) and FOR4 (bottom) (current density of 60 mA g^−1^, potential window 0.005–3 V, recorded at room temperature). Reproduced with permission from ref. 195. Copyright 2010, Royal Society of Chemistry. (C) Galvanostatic cycling performance of Li–O_2_ batteries using Mn-MOF-74, Co-MOF-74, and MnCo-MOF-74 electrodes at a limited capacity of 1000 mA h g^−1^ with a current density of 200 mA g^−1^. End voltage *vs.* cycle number of the electrodes. Reproduced with permission from ref. 198. Copyright 2018, American Chemical Society.

MOFs with large surface areas, adjustable pore structures, and redox metal centres are promising electrode materials for electrochemical capacitors and rechargeable batteries.^14,182–186^ However, the inherent poor electrical conductivity of MOFs is one of the greatest obstacles to achieve high performance in capacitors. In bimetallic MOFs, the electrochemical properties can be tuned by mixing two different metals in the SBUs. Bimetallic MOFs with enhanced electrical conductivity have been applied as the electrode materials for supercapacitors.^187–193^ Pei, Chen and co-workers improved the electrical conductivity of MOFs by partially exchanging Ni^2+^ in the Ni-MOF ([Ni_3_(OH)_2_(tp)_2_(H_2_O)_4_]·2H_2_O, tp = C_8_H_4_O_4_^2−^) with Co^2+^ or Zn^2+^ for hybrid supercapacitors.^190^ The specific capacities of Co/Ni-MOF, Zn/Ni-MOF and Ni-MOF were 236.1, 161.5 and 142.3 mA h g^−1^ at 1 A g^−1^, with a capacity retention of 82.8%, 56% and 37% at 10 A g^−1^, respectively (Fig. 14A). The assembled hybrid supercapacitors (Co/Ni-MOF//CNTs–COOH) exhibited an energy density of 49.5 W h kg^−1^ and a power density of 1450 W kg^−1^. The metal ion exchange resulted in an increase of free holes and the interlayer distance and in turn led to an enhancement of the electrical conductivity and surface area, explaining the enhanced performance of bimetallic MOFs for capacitors.

Bimetallic MOFs have been investigated as anode and cathode materials for lithium-ion batteries (LIBs). When using bimetallic MOFs as anode materials, the adjustable pores can allow Li ions to be stored and reversibly inserted/extracted, leading to insertion-type electrodes.^194^ Some bimetallic MOFs enabling reversible transformation/regeneration can be employed as conversion-type electrodes.^195^ For example, Vittal *et al.* investigated the electrochemical performance of FOR4 (Zn_1.5_Co_1.5_(HCOO)_6_).^195^ The formate-bridged MOFs were converted to lithium formate MOFs upon lithiation and regenerated upon de-lithiation. The reversible conversion reaction led to good cycling stability of FOR4 (Fig. 14B), with a high reversible capacity of 510 mA h g^−1^ up to 60 cycles at 60 mA g^−1^, exceeding that of monometallic CoMOF (FOR3). As cathodes in LIBs, bimetallic MOFs with redox metal centres and good Li-ion mobility within the solid are good candidates, such as K_0.14_Mn_1.43_[Fe(CN)_6_]·6H_2_O^196^ and K_0.1_Ni[Fe(CN)_6_]_0.7_ [Fe(CN)_6_]_0.3_·4.7H_2_O.^197^

Bimetallic MOFs can be applied as cathode materials for lithium–oxygen batteries (LOBs). The porous structures and tuneable bimetallic active sites endow bimetallic MOFs with the capabilities to optimize the performance. Lee and co-workers used bimetallic MnCo-MOF-74 materials as cathode catalysts for Li–O_2_ batteries.^198^ MnCo-MOF-74 delivered a discharge capacity of 11 150 mA h g^−1^ and excellent cyclability (44 cycles) with a low overpotential at a limited capacity of 1000 mA h g^−1^, outperforming the monometallic counterparts, Mn- and Co-MOF-74 (Fig. 14C). The synergistic integration of Mn- and Co-metal clusters contributed to the improved efficiency and reversibility.

### 3.4 Luminescence sensing

Luminescent MOFs (LMOFs) are an important sub-category of MOFs.^199^ Lanthanide (Ln) cations are particularly attractive for the construction of LMOFs because of their characteristic sharp emission bands and high quantum yields in the near-infrared and visible regions. Typically, Eu^3+^ and Tb^3+^ are usually used in LnMOFs because of their strong characteristic red emission at 614 nm (^5^D_0_ → ^7^F_2_) and green emission at 541 nm (^5^D_4_ → ^7^F_5_), respectively.

Mixed Ln^3+^ ions with different emissions can be integrated into a MOF framework to tune the luminescence properties.^200^ Moreover, the metal distribution (solid solution or core–shell) in mixed LnMOFs influences the luminescence properties. Mahon, Burrows and co-workers studied the effect of metal distribution on the luminescence properties of mixed LnMOFs.^201^ Solid solution mixed LnMOFs, [Gd_0.17_Tb_0.19_Eu_0.64_(Hodip) (H_2_O)]·*n*H_2_O (H_4_odip = 5,5′-oxydiisophthalic acid), showed an emission spectrum with strong bands of Eu^3+^ and weak bands of Tb^3+^ and H_4_odip. This is due to the energy transfer from Tb^3+^ to Eu^3+^, resulting in a quenching of Tb^3+^ transitions. Core–shell mixed LnMOFs displayed different emission spectra because the metal energy transfer between Ln^3+^ ions was prevented. Gd@Tb@Eu and Tb@Eu@Gd showed emission spectra dominated by Tb^3+^ emissions with lower intensity Eu^3+^ and ligand transitions. Eu@Gd@Tb exhibited an emission spectrum with only Eu^3+^ and ligand transitions present. In all the core–shell structures, the core Ln^3+^ emissions were not observable. Therefore, alteration of shell ordering and thickness could tailor the luminescence properties of core–shell mixed Ln-MOFs.

A variety of bimetallic LnMOFs have acted as ratiometric sensors with high sensitivity and high selectivity toward ionic species, pH, temperature, environmental toxins, explosives, biomolecules, *etc.*^202–204^

**Fig. 15:**
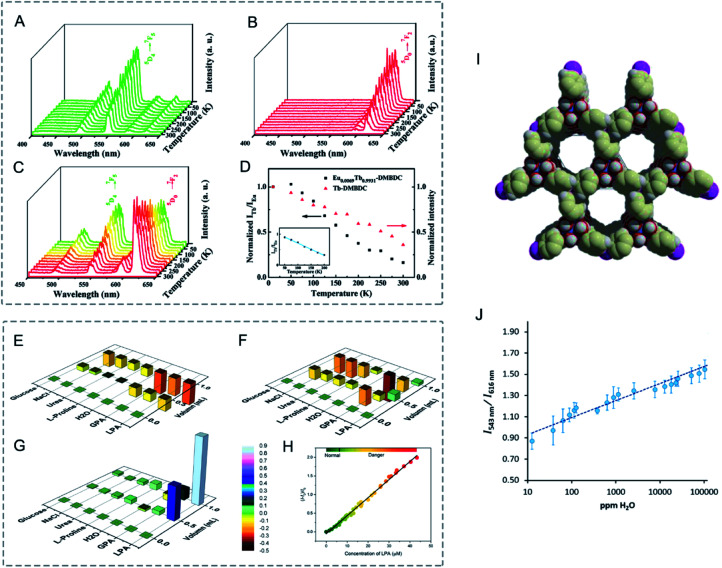
(A–C) Emission spectra of (A) **Tb-DMBDC**, (B) **Eu-DMBDC**, and (C) **Eu0.0069Tb0.9931-DMBDC** recorded between 10 and 300 K (excited at 355 nm from the third harmonic of a Nd:YAG laser). (D) Temperature dependence of the integrated intensity ratio of Tb^3+^ (545 nm) to Eu^3+^ (613 nm) for **Eu0.0069Tb0.9931-DMBDC** (black squares), and temperature dependence of the integrated intensity of Tb^3+^ (545 nm) for **Tb-DMBDC** (red triangles). (Inset) Fitted curves of the integrated intensity ratio for **Eu0.0069Tb0.9931-DMBDC** from 50 to 200 K. Reproduced with permission from ref. 206. Copyright 2012, American Chemical Society. (E–G) Relative luminescence intensity changes of (E) **Tb-ZMOF**, (F) **Eu-ZMOF**, and (G) **MZMOF-3** in the presence of different analytes. The intensity change is indicated by the colormap from −0.5 to 0.9. (H) Integrated luminescence intensity changes of **MZMOF-3** suspended in MeOH toward 0.03, 0.10, and 0.13 mM LPA. Reproduced with permission from ref. 207. Copyright 2015, American Chemical Society. (I) Chemical Structure of PCM-22. (J) Relative photoemission response ratios obtained upon addition of trace H_2_O to Eu_1_ : Tb_5_-PCM-22 pre-soaked in D_2_O showing a linear response. Error bars were obtained from three separate experiments. Reproduced with permission from ref. 208. Copyright 2017, Elsevier.

Bimetallic LnMOFs with dual emissions can be employed as self-referencing luminescence thermometers based on the intensity ratios of two separate transitions.^205^ Qian, Chen and co-workers reported luminescent mixed Ln-MOFs, (Eu_*x*_Tb_1−*x*_)_2_(DMBDC)_3_(H_2_O)_4_·DMF·H_2_O (Eu_*x*_Tb_1−*x*_–DMBDC, DMBDC = 2,5-dimethoxy-1,4-benzenedicarboxylate, *x* = 0.0011, 0.0046, and 0.0069) as luminescent thermometers.^206^ From 10 to 300 K, the luminescence intensity of Tb^3+^ at 545 nm in Tb-DMBDC and Eu^3+^ at 613 nm in Eu-DMBDC both decreased gradually as the temperature increases (Fig. 15A and B). The mixed lanthanide MOFs exhibited a significantly different temperature-dependent luminescence behaviour. In Eu_0.0069_Tb_0.9931_–DMBDC, the emission intensity of Tb^3+^ decreased, while that of Eu^3+^ increased with the increase of temperature (Fig. 15C). The emission intensity ratio (*I*_Tb_/*I*_Eu_) correlated well with the temperature in the range of 50 to 200 K (Fig. 15D). Moreover, Eu_0.0069_Tb_0.9931_–DMBDC emitted temperature dependent luminescence colours from green-yellow to red at temperatures ranging from 10 to 300 K. The exhibited temperature-dependent emissions and luminescence colours in Eu/Tb-DMBDC may be attributed to the temperature-dependent energy transfer from Tb^3+^ to Eu^3+^ ions.

Shi, Zaworotko and co-workers synthesized mixed Ln zeolite-like MOFs (Ln-ZMOFs), Eu_*x*_Tb_1−*x*_-ZMOF, as a fluorescent indicator for lysophosphatidic acid (LPA), a cancer biomarker.^207^ In Eu_0.6059_Tb_0.3941_-ZMOF (MZMOF-3), the integrated intensity ratio of Eu^3+^ and Tb^3+^ (*I*_Eu_/*I*_Tb_) correlated well with the concentration of LPA in MeOH (Fig. 15H). Moreover, MZMOF-3 exhibited selective detection for LPA over additives (water, glucose, Na^+^, Cl^−^, proline, and urea). For MZMOF-3, the luminescence intensity of LPA was 10–80 times higher than those of the additives (Fig. 15G). However, Tb-ZMOF and Eu-ZMOF exhibited similar fluorescence emission signals towards additives and LPA (Fig. 15E and F).

Mixed Ln-MOFs have also been studied as sensors for quantitative detection of water and identification of solvents. Humphrey and co-workers synthesized mixed-metal PCM-22 with controlled amounts of Eu^3+^, Gd^3+^ and Tb^3+^ as sensors for the identification of a wide range of solvents, and especially for trace H_2_O detection in D_2_O (Fig. 15I and J).^208^ PCM-22, with a formula of [Ln(tctp) (OH_2_)_3_]·3(1,4-dioxane), was synthesized by the reaction of Ln(NO_3_)_3_ with P(C_6_H_4_-*p*-CO_2_H)_3_ (tctpH_3_). For binary Eu_1_ : Tb_1_-PCM-22, based on the emissions of Tb^3+^ (616 nm) and Eu^3+^ (543 nm), the ratio of *I*_543_/*I*_616_ showed a linear relationship with H_2_O concentration in D_2_O over the range of 10–120 000 ppm. For ternary PCM-22 with four Eu : Gd : Tb compositions, they provided a unique “eight-factor” fingerprint for rapid identification of 18 solvents.

In conclusion, due to the synergistic effect between the two metals, bimetallic MOFs can exhibit significantly enhanced physical and chemical features compared with their monometallic counterparts. Therefore, through the rational design of bimetallic MOFs, they can afford excellent performance in gas adsorption, catalysis, energy storage and conversion, luminescence sensing, and so on.

## 4. Synthesis of bimetallic MOF derivatives

Since the MOF pyrolysis was reported by Xu and co-workers for porous carbon synthesis in 2008^209^ and for metal oxide synthesis in 2010,^210^ MOF derivatives have been intensively designed and synthesized.^33,211^ Especially, bimetallic MOFs with controllable compositions in the SBUs provide a promising platform for the preparation of a variety of functional materials, such as carbon composites (carbon supported atomically dispersed metals, metal NPs, alloy, oxides, carbides, sulphides, and phosphides), metal compounds (oxides, hydroxides, nitrides, sulphides, and phosphides), and MOF composites, which have a wide variety of physicochemical properties and applications.

### 4.1 Carbon composites

The pyrolysis of MOFs has been adopted as an effective way to obtain carbon composites with various morphologies and compositions. The texture and graphitization degree of carbon supports have a great influence on the performance of the composites. Carbon supports with a high surface area and hierarchical pores can guarantee the free diffusion of substrates.^212^ Graphitized carbon can enhance electrical conductivity and chemical and thermal stability.

**Fig. 16:**
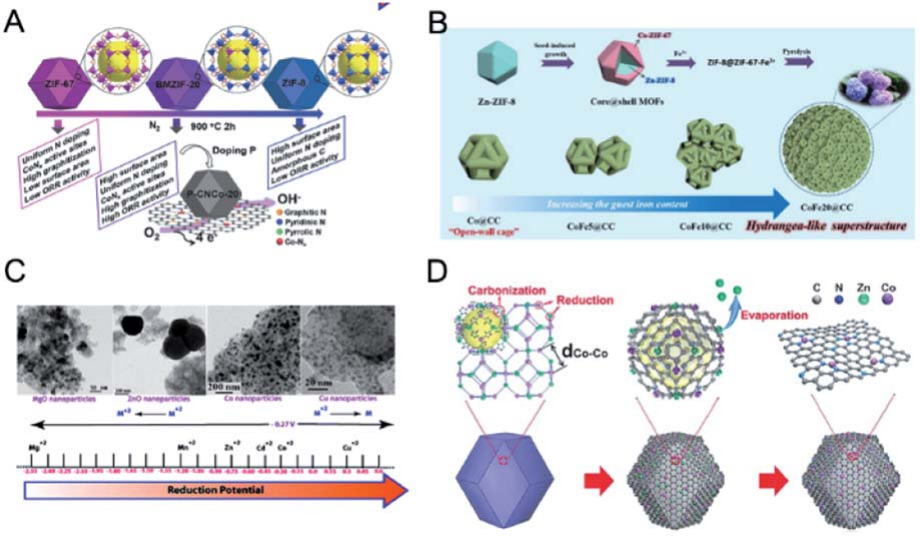
(A) Schematic illustration for the preparation of porous carbons from BMZIFs. Reproduced with permission from ref. 218. Copyright 2015, Wiley-VCH. (B) Schematic illustration of the construction of the nature-inspired hydrangea-like superstructure of open carbon cages *via* morphology-controlled thermal transformation of core@shell MOFs. Reproduced with permission from ref. 219. Copyright 2019, Wiley-VCH. (C) The effect of reduction potential of metal atoms present in the MOFs on the formation of metal/metal oxide nanoparticles. Metals having a reduction potential above −0.27 V undergo thermolysis in an N_2_ atmosphere to give pure metal nanoparticles, whereas metals with a reduction potential less than −0.27 V, even in an N_2_ atmosphere, produce metal oxides only. Reproduced with permission from ref. 223. Copyright 2012, Royal Society of Chemistry. (D) The formation of Co SAs/N-C. Reproduced with permission from ref. 236. Copyright 2016, Wiley-VCH.

ZIFs have been widely investigated as precursors to synthesize carbon-based materials. The pyrolysis of bimetallic ZIFs can realize well-graphitized carbons, high-surface-area carbons, and active metal–N_*x*_–C sites at the same time, which cannot be achieved by monometallic ZIFs. For example, the pyrolysis of Zn-based ZIF-8 can afford high-surface-area carbon with high N contents but fail to provide active metal–N_*x*_–C sites and well-graphitized carbon.^213,214^ On the other hand, the pyrolysis of Co-based ZIF-67 can give well-graphitized carbon and highly active Co–N_*x*_–C sites but only offer carbon with a low surface area and porosity.^215–217^ In this regard, Xiong, Jiang and co-workers employed a series of bimetallic ZIFs (BMZIFs-*n*, *n* represents the ratio of Zn/Co) as precursors to afford Co, N-doped porous carbons (CNCo-*n*) with a large surface area, high graphitization degree, and highly dispersed N and CoN_*x*_ active species (Fig. 16A).^218^ BMZIFs-*n* were pyrolyzed at 900 °C under N_2_, during which the generated ZnO was reduced by carbon and then evaporated. After treating the calcined products in H_2_SO_4_ at 80 °C to remove the inactive and unstable Co species, CNCo-*n* could be afforded. The BET surface areas of CNCo-*n* (*n* = 0, 0.2, 5, 10, 20, 40, and 100) were 270, 362, 550, 1090, 1225, 1480, and 2184 m^2^ g^−1^, respectively. Besides, Yamauchi and co-workers thermally transformed core–shell ZIF-8@ZIF-67 into nanoporous nitrogen-doped carbon@graphitic carbon (NC@GC) materials.^99^ ZIF-8@ZIF-67(*x*) (*x* represents the molar ratio of Co^2+^/Zn^2+^) materials were thermally treated at 800 °C under a N_2_ flow, followed by washing with HF solution to remove the residual Zn and unstable Co species. The obtained NC@GC samples showed intermediate surface areas between those of NC and GC derived from ZIF-8 and ZIF-67, respectively. In addition, NC@GC showed mesopores on the particle surface and abundant layered graphitic carbon structures, which were similar to those of GC.

Recently, Xu and co-workers reported that introducing guest Fe ions into a ZIF-8@ZIF-67 precursor led to the self-assembly of open carbon cages into a hydrangea-like 3D superstructure (Fig. 16B).^219^ Direct pyrolysis of core–shell ZIF-8@ZIF-67 produced isolated open-wall N-doped carbon cages with insufficient contact and total conductivity. The introduction of guest Fe ions into the MOF precursor formed FeCo alloy NPs during the pyrolysis, which could catalyse the growth of carbon nanotubes and thus interconnect neighbouring cages to form a hydrangea-like 3D superstructure.

The employment of bimetallic MOFs as precursors can afford precise size, composition, and structure control of the supported metal/oxide NPs.^220–222^ This is benefited from the ability to control the compositions and distributions of metal ions in MOFs, which are not achievable by other synthetic methods. The calcination of bimetallic MOFs under an inert atmosphere can afford metal alloy or oxide NPs depending on the reduction potential of the metal ions.^223^ Metal ions (*e.g.*, Cu^2+^ and Co^2+^) with a reduction potential of −0.27 V or higher usually form metal NPs after calcination under an inert atmosphere, whereas metal ions (*e.g.*, Cd^2+^ and Mg^2+^) with reduction potentials lower than −0.27 V form metal oxide NPs (Fig. 16C). For example, Chen and co-workers calcined bimetallic MOFs (Fe_3_[Co(CN)_6_]_2_) at 600 °C in N_2_, affording FeCo alloy NPs encapsulated in N-doped graphene layers.^220^ Gao, Lou and co-workers pyrolyzed Co/Mn-MIL-100 at 800 °C in a 5% H_2_/Ar atmosphere, affording a MnO/Co hybrid supported by porous graphitic carbon.^224^

The type of metal compound generated from the pyrolysis of bimetallic MOFs can be affected by the elements in the ligands. Carbon atoms can diffuse into the interstices of metal atoms to form bimetallic carbides.^225–227^ For example, Su and co-workers reported the synthesis of Co/Mo_2_C/Mo_3_Co_3_C@C and Ni/Mo_2_C@C derived from two bimetallic MOFs (CoMo-MOF and NiMo-MOF) annealed at 900 °C in N_2_, respectively.^226^ Other metal compounds (metal nitrides, phosphides and sulphides) supported on carbon materials can also be synthesized from bimetallic MOFs if suitable ligands with heteroatoms are employed.

Bimetal nitrides, phosphides and sulphides embedded in carbon can also be obtained through introducing additional precursors (N/P/S-containing regents) before^228–231^ or after the pyrolysis process.^94,232–235^ Moreover, additional metal precursors can be introduced into MOFs before calcination to alter the composition of metal compounds.

The pyrolysis of MOFs serves as an ideal route for preparing various metal single atoms (SAs) on porous carbon (SAs/C). Delicate control is required to avoid the conversion of metal ions into aggregated NPs at high temperature. Bimetallic MOFs offer a promising platform for the synthesis of SAs/C based on the idea of using a second metal that is easily removed after pyrolysis to extend the distance between adjacent targeted metals to avoid aggregation during pyrolysis.^236–240^ Wu, Li and co-workers demonstrated the concept for the synthesis of Co SAs on N-doped porous carbon (Co SAs/N–C) through the pyrolysis of a bimetallic Zn/Co MOF (Zn/Co-BMOF).^236^ The addition of Zn^2+^ could dilute the concentration of Co^2+^ and extend the adjacent distances between Co atoms. During the pyrolysis process, Zn was evaporated at high temperature, leaving abundant N sites to anchor and stabilize the isolated Co atoms (Fig. 16D). Co SAs/N-C obtained by calcination at 800 and 900 °C showed a 4 and 2 metal coordination number with surrounding N atoms, respectively. Further increasing the calcination temperature to 1000 °C broke the Co–N bonds and caused the formation of Co NPs. In the sequent work, Wu, Li and co-workers synthesized Fe–Co dual sites embedded on N-doped porous carbon [(Fe,Co)/N–C] derived from Zn/Co-BMOF encapsulated FeCl_3_.^241^ FeCl_3_ was immobilized within the pores of Zn/Co-BMOF by a double-solvent method. During the calcination, Fe salts were reduced by carbon and bonded with neighbouring Co atoms to form Fe–Co dual-sites. The Fe species would accelerate the decomposition of MOFs and force the generation of voids inside the N-C support.

### 4.2 Metal compounds

The thermal calcination of bimetallic MOFs in air can lead to their decomposition into their corresponding bimetallic oxides. To achieve a higher surface area and porosity and preserve the original structure of parent MOFs to a certain extent, a two-step annealing (first in N_2_ and then in air) can be applied to synthesize metal oxide nanostructures. The use of bimetallic MOFs as precursors to obtain bimetallic oxides has emerged as a powerful synthetic route, offering the possibility to precisely control the compositions and structures of metal oxides.

**Fig. 17:**
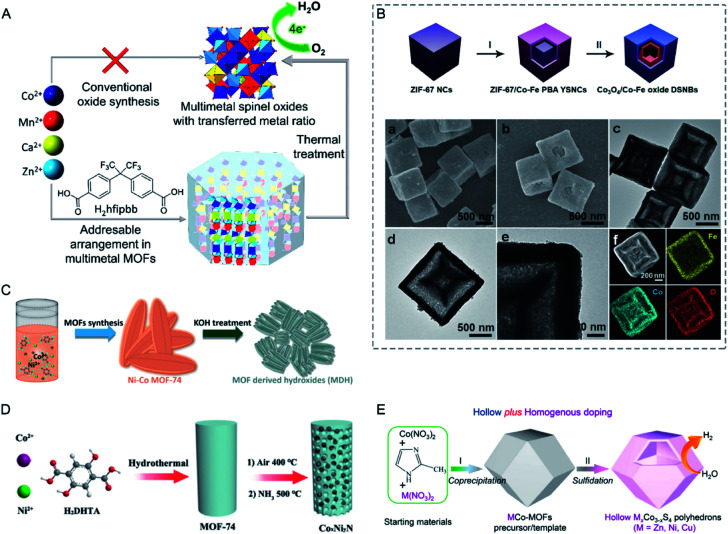
(A) Thermal decomposition of multimetal MOFs allows the obtaining of multication oxides with compositions not attainable with conventional oxide synthetic routes. The metal ratios in the resulting oxides are transferred from their corresponding parent MOFs. Reproduced with permission from ref. 244. Copyright 2019, American Chemical Society. (B) Schematic illustration of the formation process of Co_3_O_4_/Co–Fe oxide DSNBs: (I) ion–exchange reaction between ZIF-67 NCs and [Fe(CN)_6_]^3−^ ions to form ZIF-67/Co–Fe PBA YSNCs and (II) subsequent conversion to Co_3_O_4_/Co–Fe oxide DSNBs by thermal annealing (top). (a, c) FESEM and (b, d–f) TEM images of (a, b) ZIF-67 NCs and (c–f) ZIF-67/Co–Fe PBA YSNCs. Reproduced with permission from ref. 248. Copyright 2018, Wiley-VCH. (C) Illustration of the synthesis process from MOF-74 to Ni–Co MDH. Reproduced with permission from ref. 250. Copyright 2017, American Chemical Society. (D) Schematic diagram of synthesis for porous Co_*x*_Ni_*y*_N composites. Reproduced with permission from ref. 253. Copyright 2019, American Chemical Society. (E) Schematic illustration of fabrication of hollow Co-based bimetallic sulphides. Reproduced with permission from ref. 254. Copyright 2016, American Chemical Society.

The achieved success in the control of metal distribution in MOFs can allow the tuning of the composition of the obtained metal oxides.^242–245^ Zhou and co-workers synthesized Zn_*x*_Co_3−*x*_O_4_ (0 < *x* ≤ 1) hollow polyhedra through calcination of bimetallic Zn-Co-ZIFs.^242^ Bimetallic Zn–Co-ZIFs were first heated in N_2_ and then in air at 400 °C. The first thermal treatment in N_2_ was of vital importance to partially preserve the porous structure of ZIFs. Monge, Gándara and co-workers demonstrated the control of the composition of multimetal oxides through the use of MOFs constructed from up to four different metal elements (Fig. 17A).^244^ Multimetallic MOFs, constructed from a helical inorganic SBU with different cations (Zn^2+^, Co^2+^, Mn^2+^, and Ca^2+^) and an organic linker H_2_hfipbb [4,4′-(hexafluoroisopropylidene)bis-(benzoic acid)], were subjected to a thermal treatment in air at 800 °C to obtain the corresponding multimetal oxides. The metal arrangements and ratios of MOFs could be translated to metal oxides after calcination.

Metal oxides with complex structures can be fabricated from rationally designed bimetallic MOF precursors.^246,247^ Song, Lou and co-workers reported the preparation of Co_3_O_4_/Co–Fe oxide double-shelled nanoboxes (DSNBs) derived from ZIF-67/Co–Fe PBA yolk–shell nanocubes (YSNCs).^248^ ZIF-67/Co–Fe PBA YSNCs were fabricated through an anion-exchange reaction between ZIF-67 and [Fe(CN)_6_]^3−^ ions. The ZIF-67/Co–Fe PBA YSNCs were further converted to Co_3_O_4_/Co–Fe oxide DSNBs by calcination in air (Fig. 17B).

Metal hydroxides can be fabricated from bimetallic MOF precursors through alkali hydrolysis.^249–252^ Zou, Liu and co-workers reported the synthesis of bimetallic double hydroxides (MDHs) derived from bimetallic NiCo-MOF-74 through an alkaline treatment (Fig. 17C).^250^ Ni/Co MOF-74 samples were dispersed in a 2 M KOH aqueous solution under stirring at room temperature, followed by hydrothermal treatment at 120 °C for 2 h. The hydrothermal treatment with KOH resulted in the destruction of micron-sized MOF-74 and the formation of nanoscale MDH.

Bimetallic nitrides can be prepared from bimetallic MOFs through nitridation under an NH_3_ atmosphere. Guo and co-workers synthesized bimetallic CoNi nitrides derived from bimetallic CoNi-MOF-74.^253^ CoNi-MOF-74 was first converted to Ni_1_Co_2_O_4_ through calcination at 400 °C in air, and then transformed into Co_2_Ni_1_N by nitridation under an NH_3_ atmosphere at 500 °C (Fig. 17D). The resulting metal nitrides inherited the morphology from their corresponding precursors.

Bimetallic sulphides can be synthesized using bimetallic MOFs as templates *via* a hydrothermal sulfidation reaction.^193,254–256^ Zou and co-workers reported the synthesis of hollow bimetallic sulphides (M_*x*_Co_3−*x*_S_4_, M = Zn, Ni, and Cu) by solvothermal sulfidation and thermal annealing from Co-based bimetallic MOFs.^254^ MCo-MOFs (M = Zn, Ni, and Cu) were treated in an ethanol solution of thioacetamide (TAA) at 120 °C for 4 h, followed by calcination under a N_2_ atmosphere at 350 °C for 2 h (Fig. 17E). The obtained M_*x*_Co_3−*x*_S_4_ preserved the rhombic dodecahedral morphology of MOF precursors, while formed a hollow structure after the sulfidation procedure. S^2−^ ions released from TAA first reacted with metal ions on the MOF surface to form a thin layer of sulphides, which could promote the outward diffusion of metal ions against the inward diffusion of S^2−^ ions. Then the inner framework was gradually dissolved and released metal ions to react with S^2−^ ions on the outer surface, which finally led to the generation of a hollow void inside the shell.

Transition metal phosphides (TMPs) show extremely low cost and expected long-term stability in both acidic and alkaline operating environments. Bimetallic MOFs have been applied for the synthesis of TMPs.^257–262^ Zhao and co-workers synthesized nickel–cobalt mixed metal phosphide nanotubes (Co_*x*_Ni_*y*_P, *x* and *y* represent the molar ratios of Co and Ni in the MOF precursor, respectively) derived from bimetallic CoNi-MOF-74.^258^ Bimetallic MOF-74 materials with different Co/Ni ratios were first calcined at 350 °C in air to afford Co_*x*_Ni_*y*_O, followed by phosphorization with NaH_2_PO_2_ at 300 °C in N_2_ to afford Co_*x*_Ni_*y*_P.

As summarized above, various types of metal compounds can be obtained through the treatment of bimetallic MOFs.

### 4.3 MOF composites

**Fig. 18:**
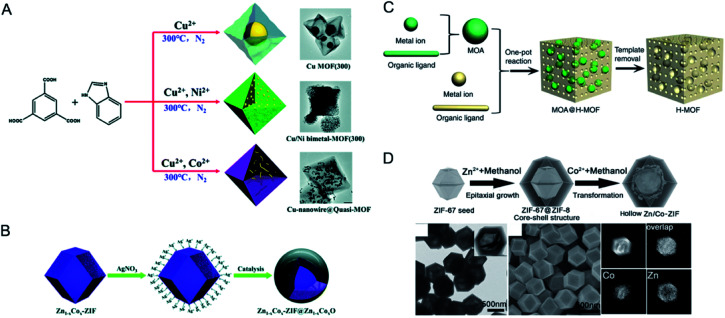
(A) Scheme and TEM images of Cu MOF(300), Cu/Ni bimetal-MOF(300), and Cu-nanowire@Quasi-MOF. Reproduced with permission from ref. 264. Copyright 2019, Royal Society of Chemistry. (B) Synthetic procedure of the Zn_1−*x*_Co_*x*_-ZIF@Zn_1−*x*_Co_*x*_O hybrid photocatalyst. Reproduced with permission from ref. 266. Copyright 2018, Royal Society of Chemistry. (C) *In situ* self-assembly of MOA through the reaction between a metal ion and organic ligand. MOA@H-MOF composite formed by a one-pot self-assembly reaction. H-MOF formed through removing the MOA template. Reproduced with permission from ref. 267. Copyright 2015, Nature Publishing Group. (D) Ilustration of the structural evolution (top). TEM image, SEM image, and EDX mappings of H-Zn/Co-ZIF (bottom). Reproduced with permission from ref. 268. Copyright 2015, Wiley-VCH.

Complete decomposition of MOFs usually leads to a dramatic decrease of the surface area and loss of well-defined pore structures.^209,213^ Recently, Xu and co-workers have demonstrated a new concept of “quasi-MOFs” fabricated through controlled deligandation of MOFs, which can realize both a porous structure and exposed inorganic nodes.^263^ This controlled thermal transformation method has been successfully applied on solid solution bi- and multi-metallic MOFs for the synthesis of MOF immobilized metal NPs or oxides.^264,265^ This design takes advantage of different coordination abilities of the metal ions with the organic linkers. The metal ions with weaker coordination with the organic linkers transform into metal NPs/compounds under certain conditions while the other metal ions remain coordinated with the organic linkers to preserve the framework. Hu and co-workers reported a thermal transformation of a benzimidazole-modified Cu/Co bimetal-MOF into a Cu-nanowire@quasi-MOF composite (Fig. 18A).^264^ The Cu/Co bimetal-MOF was calcined at 300 °C for 30 min under a N_2_ atmosphere to afford a quasi-MOF matrix with a partially preserved MOF structure embedded with Cu nanowires. Under similar calcination conditions, monometallic Cu-MOF transformed into core–shell Cu_2_O@N doped carbon composites. During the calcination process, Cu-ligand bonds with weaker coordination would break and form metastable Cu NPs, while the Co-MOF part with higher stability would serve as a morphology retainer and a nano-channel template to reassemble Cu NPs into Cu nanowires.

Besides thermal transformation, solvent induced transformation is also a powerful strategy for the conversion of bimetallic MOFs to composites of MOFs and metal compounds. Luo and co-workers obtained Zn_1−*x*_Co_*x*_-ZIF@Zn_1−*x*_Co_*x*_O through the transformation of Zn_1−*x*_Co_*x*_-ZIF with AgNO_3_ (Fig. 18B).^266^ Zn_1−*x*_Co_*x*_-ZIF was dispersed in an aqueous solution of silver nitrate (AgNO_3_) and stirred for 1 h to obtain Zn_1−*x*_Co_*x*_-ZIF@Zn_1−*x*_Co_*x*_O. AgNO_3_ acted as the catalyst to break the coordinative bonds of MOFs and created a hydroxyl rich environment for the formation of Zn_1−*x*_Co_*x*_O on the surface of Zn_1−*x*_Co_*x*_-ZIF.

Selectively removing the core MOFs in single- or multi-core–shell bimetallic MOFs can obtain hierarchical-pore or hollow MOF immobilized metal compound composites. In this design, less stable MOFs act as seeds to grow stable MOFs as the shell, followed by selective removal of the core MOFs to generate pores. The generated mesopores/macropores can facilitate the diffusion of substrates and the desorption of products.

For example, Li, Zhong and co-workers used MOF-5 as a template to grow UiO-66(Zr), followed by the removal of the cores to synthesize hierarchical UiO-66(Zr) (H-UiO-66).^267^ MOF-5 is stable in certain solvents but sensitive towards moisture and acid, while UiO-66(Zr) is stable even in acidic solutions. Nano-sized MOF-5 particles were first synthesized, followed by the growth of UiO-66(Zr). The obtained material was washed with acid aqueous solution to get the targeted material H-UiO-66(Zr) with Zr species anchoring in the mesopore surface (Fig. 18C). In another system, Wu, Li, and co-workers reported a mild phase transformation of core–shell ZIF-67@ZIF-8 structures into hollow Zn/Co ZIF particles.^268^ They treated core–shell ZIF-67@ZIF-8 with Co^2+^ in methanol at 120 °C for 4 h, resulting in the dissolution of the solid interior and then the formation of a hollow structure with interlaced nanoplates (Fig. 18D). The authors proposed that methanol molecules formed H-bonds in the presence of Co^2+^ to break the coordination bonds between Co^2+^ and 2-MeIm, driving the structural evolution of ZIF-67. Muhler, Fischer and co-workers reported similar results in parallel.^269^ During the epitaxial growth of ZIF-8 on preformed ZIF-67 under solvothermal conditions, ZIF-67 partially dissolved to release cobalt ions to form cobalt hydroxide with a sheet-like structure.

Carbon composites, metal compounds and MOF composites can be derived from bimetallic MOFs through pyrolysis, hydrothermal treatment, and so on. This wide variety of MOF-derived materials can be applied in many applications, which will be introduced in the following section.

## 5. Applications of bimetallic MOF derivatives

The applications of the majority of bimetallic MOFs are restricted by their low stability, poor conductivity and blockage of active sites. Bimetallic MOF-derived materials show high stability, high conductivity, and exposed active sites. These properties provide bimetallic MOF derivatives with more opportunities for applications in catalysis under harsh conditions and electrochemical energy storage and conversion.

### 5.1 Catalysis

#### 5.1.1 Organocatalysis

Diverse MOF-derived porous materials are very promising for various organic reactions, including reduction, oxidation, dehydrogenation, and biomass transformation.

**Fig. 19:**
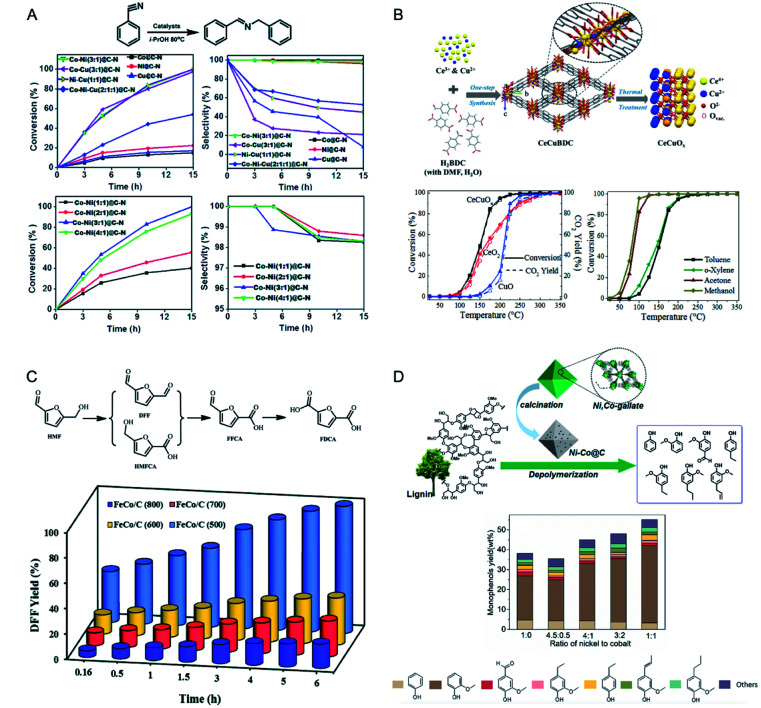
(A) The conversion of benzonitrile and selectivity of the corresponding imine with various catalysts for the transfer hydrogenation of benzonitrile. Reaction conditions: 0.5 mmol nitriles, 4 mL i-PrOH, catalyst (metal 10 mol%), 80 °C, and 15 h. Reproduced with permission from ref. 221. Copyright 2016, Royal Society of Chemistry. (B) Schematic diagram of the synthesis procedure of the bimetallic MOF-derived CeCuO_*x*_ catalyst (top). Toluene conversion and CO_2_ yield *versus* temperature curves over CeO_2_, CuO, and CeCuO_*x*_ catalysts (left bottom). Conversion *versus* temperature curves for complete oxidation of methanol, acetone, toluene, and *o*-xylene over the CeCuO_x_ catalyst. (Note: [VOC] = 500 ppm, *m*_catalyst_ = 0.15 g, and gas hourly space velocity (GHSV) = 23 000 h^−1^) (right bottom). Reproduced with permission from ref. 278. Copyright 2019, Wiley-VCH. (C) Products of HMF oxidation (top). DFF yield as a function of reaction time over the FeCo/C catalysts. Reaction conditions: 1 mmol HMF, catalyst (metal 20 mol%), Na_2_CO_3_ 1 mmol, 2 mL toluene, 1 MPa O_2_, 100 °C, and 6 h (bottom). Reproduced with permission from ref. 290. Copyright 2016, Royal Society of Chemistry. (D) Depolymerization of renewable lignin extracted from poplar to monophenolic compounds with the Ni–Co@C catalyst was prepared from MOFs (top). Monophenol yield on the Ni_*x*_Co_1−*x*_/C catalyst (bottom). Reproduced with permission from ref. 294. Copyright 2019, American Chemical Society.

MOF-derived materials are active for many reduction reactions, such as the hydrogenation of phenol,^270^ nitro compounds^271,272^ and nitriles,^221^ and the reduction of NO^273,274^ and CO_2_.^275^ As an example, Li and co-workers applied bimetallic alloy NPs embedded in an N-doped carbon matrix (M–M′@C–N, M/M′ = Co, Ni, Cu) derived from bimetallic MOFs (M–M′(1,4-bdc)_2_(dabco)·4DMF·1/2H_2_O, M/M′ = Co, Ni, Cu) for transfer hydrogenation of nitriles to imines.^221^ In the hydrogenation of benzonitrile to the corresponding imine, Co–Ni(3 : 1)@C–N exhibited remarkable activity with over 98% yield for the desired product after 15 h, which was 5 times and 7 times as high as that of Ni@C–N and Co@C–N, respectively (Fig. 19A). Co–Ni@C–N with a Co–Ni molar ratio of 3 : 1 showed the highest activity; further decreasing or increasing the proportion of Co resulted in a decrease of activity.

Bimetallic MOF-derived porous catalysts have also been developed for a variety of oxidation reactions, such as the oxidation of alcohols,^271,276,277^ acetone, olefin and toluene,^278^ and organic pollutant degradation.^279,280^ As an example, Han, Yeung and co-workers prepared a binary metal oxide (CeCuO_*x*_) derived from bimetallic MOFs (CeCuBDC) as the catalyst for the oxidation of organic compounds.^278^ In the oxidation of toluene to CO_2_, CeCuO_*x*_ exhibited high catalytic activity, with a *T*_50_ (temperature required for 50% conversion) and *T*_90_ of 150 and 186 °C, respectively (Fig. 19B). CeCuO_x_ showed lower activation energy (52.4 kJ mol^−1^) than CeO_2_ (57.5 kJ mol^−1^) and CuO (70.5 kJ mol^−1^). The CeCuO_x_ catalyst was also highly active for the oxidation of methanol, acetone and *o*-xylene, with a *T*_50_ of 80, 87 and 142 °C, respectively.

Controlled generation of hydrogen from chemical hydrogen storage materials is of vital importance to the hydrogen-economy paradigm in the future.^281–284^ Bimetallic MOF-derived nanomaterials exhibit high catalytic performance for the release of hydrogen from chemical hydrogen storage materials such as ammonia borane (NH_3_BH_3_, AB) and lithium borohydride (LiBH_4_).^285–288^ Chen and co-workers fabricated porous cobalt phosphide supported by carbon-based nanoframeworks (CoP@CNFs), which was derived from Zn/Co-ZIF and was applied for hydrogen generation from AB.^286^ Zn/Co-ZIF was calcined in air to get Zn–Co–O@CNF, followed by phosphidation using Na_2_H_2_PO_2_ and further acid treatment to get CoP@CNFs. CoP@CNFs gave an initial TOF value of 165.5 mol_H_2__ mol_metal_^−1^ min^−1^ at 298 K, maintaining 90% of its initial activity after 4 cycles.

Biomass has been an important resource for the production of chemicals, fuels, and energy.^289^ Bimetallic MOF-derived nanocatalysts have been applied in effective biomass transformation.^290–295^ Li, Luque and co-workers applied hollow Fe–Co supported on carbon (FeCo/C) derived from bimetallic Fe/Co-MIL-45b for chemical conversions of biomass-derived platform chemicals.^290^ In the aerobic oxidation of biomass-derived 5-hydroxymethylfurfural (HMF) to 2,5-diformylfuran (DFF), FeCo/C(*T*) prepared by calcination at different temperatures (*T* = 500, 600, 700 or 800 °C) showed distinct activities. FeCo/C(500) exhibited the highest activity, with a complete conversion and >99% selectivity to DFF at 100 °C after 6 h (Fig. 19C). Jones and co-workers prepared Co/Cu–carbon (Co/Cu–C) derived from Cu/Co MOF-74 for furfural hydrogenation.^292^ The catalyst performance was investigated at 180 °C and a weight/volume flow rate (W/F) of 3.63 g_cat_ h mol^−1^, and monometallic Co–C showed high conversion (50%) and high selectivity for 2-methylfuran (49%), while Cu–C had low conversion but high selectivity toward furfuryl alcohol. CoCu–C-400 (pyrolyzed at a temperature of 400 °C) with a partial Cu shell showed reactivities characteristic of Co–C. CoCu–C-600 with a full Cu shell had reactivities similar to those of Cu–C. Although bimetallic CoCu catalysts did not show activity/selectivity improvements compared to those of monometallic catalysts for furfural conversion, these results have demonstrated synthesis–structure–property correlations, which would guide the future development of better catalysts. The conversion of lignin (10–35% by weight in biomass) to value-added chemicals, alternative fuels, and platform compounds is attractive. Bao, Ren and co-workers reported the use of bimetallic-MOF-derived Ni–Co/C materials with varying Ni/Co ratios as catalysts for lignin conversion into monophenols.^294^ In the hydrogenolysis of poplar lignin, Ni_0.5_Co_0.5_/C showed superior catalytic efficiency to Ni/C, with a yield of 55.2% to monophenols and a selectivity of 70.3% to guaiacol (Fig. 19D).

#### 5.1.2 Photocatalysis

Bimetallic MOF-derived porous nanomaterials have also been developed for photocatalysis, including photocatalytic degradation of organic pollutants, water splitting and CO_2_ reduction.

**Fig. 20:**
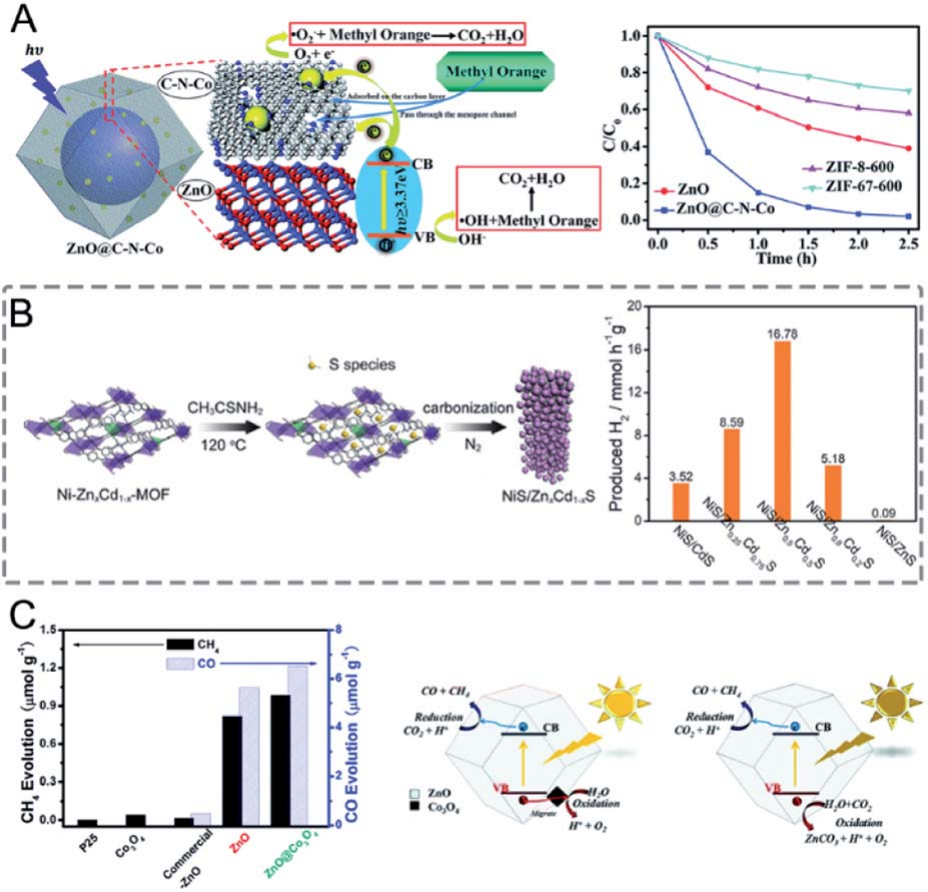
(A) Proposed mechanism of the photodegradation of organic pollutants using the as-prepared core–shell ZnO@C–N–Co as the catalyst (left). Photodegradation curves of MO over various catalysts under irradiation with UV-vis light (right). Reproduced with permission from ref. 297. Copyright 2017, Royal Society of Chemistry. (B) Schematic illustration of the synthetic procedure for NiS/Zn_*x*_Cd_1−*x*_S (left). Comparison of the photocatalytic HER rates of NiS/Zn_*x*_Cd_1−*x*_S under visible-light irradiation (right). Reproduced with permission from ref. 306. Copyright 2018, Wiley-VCH. (C) Comparison of the photocatalytic activities of the photocatalysts under UV-vis irradiation, calculated according to how each compound evolved after 6 h. Schematic illustration of the photocatalytic CO_2_ reduction with ZnO (middle) and ZnO@Co_3_O_4_ (right). Reproduced with permission from ref. 309. Copyright 2016, Royal Society of Chemistry.

Photocatalytic degradation of organic pollutants from wastewater is of ecological and environmental importance. Nanomaterials derived from bimetallic MOFs have shown high activity and stability in the degradation of organic pollutants, such as organic dyes and antibiotics.^266,279,296–299^ Li and co-workers designed ZnO@C–N–Co core–shell nanocomposites derived from Zn/Co ZIF for the degradation of methyl orange (MO).^297^ When calcining Zn/Co ZIF at 600 °C, ZnO NPs generated from the ZIF-8 shell would aggregate and move to the hollow cavity while the internal Co NPs transferred inversely to the N–C shell, resulting in the unique ZnO@C–N–Co core–shell structure. Under the irradiation of a Xe lamp (300 W) for 2.5 h, ZnO@C–N–Co exhibited a high degradation percentage of MO (99.5%), much higher than that of ZIF-67-600 (29.4%) and ZIF-8-600 (41.9%). The superior performance of ZnO@C–N–Co could be attributed to the synergistic effect between the components, in which the porous carbon shell contributed to the stabilization of ZnO and the adsorption of reactants and Co NPs inhibited the recombination of electrons and holes (Fig. 20A).

Photocatalytic water splitting for hydrogen production is a promising route to convert solar energy into renewable hydrogen energy.^300,301^ Bimetallic MOF-derived nanomaterials, such as Fe–Ni–P,^302^ CuS/ZnS,^303^ Co-doped Zn_1−*x*_Cd_*x*_S,^304^ yolk–shell CdS microcubes,^305^ and NiS/Zn_*x*_Cd_1−*x*_S,^306^ have been applied in photocatalytic water splitting for hydrogen production. As an example, Shi, Cheng and co-workers constructed non-noble metal co-catalyst/solid solution heterojunction NiS/Zn_*x*_Cd_1−*x*_S derived from Ni-Zn_*x*_Cd_1−*x*_-MOF for the production of hydrogen (Fig. 20B).^306^ Under visible-light irradiation (*λ* > 420 nm), NiS/Zn_0.5_Cd_0.5_S exhibited a high HER rate of 16.78 mmol g^−1^ h^−1^, much higher than those of NiS/ZnS (0.09 mmol g^−1^ h^−1^), NiS/ZnS (0.09 mmol g^−1^ h^−1^) and NiS/CdS (3.52 mmol g^−1^ h^−1^). The tuning of the Zn/Cd ratio in heterojunction Zn_*x*_Cd_1−*x*_S pointed to an optimized *x* of 0.5 to achieve a good balance between the light absorption capacity of the catalyst and edge of the conduction band. The co-catalyst NiS could further accelerate the water dissociation kinetics to improve the photocatalytic hydrogen evolution activity. Furthermore, constructing composites of co-catalysts and bimetallic MOF-derived photocatalysts, such as Cu_0.9_Co_2.1_S_4_@MoS_2_,^307^ CdS/Zn_*x*_Co_3−*x*_O_4_,^308^ Pt–ZnO–Co_3_O_4_,^262^ Pt–ZnS–CoS,^262^ Pt–Zn_3_P_2_–CoP,^262^ and MoS_2_@ZnCoS,^256^ can greatly enhance the activity for photocatalytic hydrogen evolution.

Photocatalytic reduction of CO_2_ is considered as an attractive approach to address fossil fuel shortage and carbon emission problems. Ye and co-workers prepared a ZnO@Co_3_O_4_ composite derived from ZIF-8@ZIF-67 for CO_2_ photoreduction to CH_4_ and CO.^309^ Under UV-vis irradiation, ZnO@Co_3_O_4_ gave a CH_4_ generation rate of 0.99 μmol g^−1^ h^−1^, higher than those of ZnO derived from ZIF-8 and Co_3_O_4_ derived from ZIF-67 (Fig. 20C). In ZnO@Co_3_O_4_, ZnO played a main role in the catalytic transformation, while Co_3_O_4_ effectively protected ZnO from photocorrosion to enhance the photocatalytic stability. Deng, Peng and co-workers used carbonized cobalt composites (C-BMZIFs) derived from bimetallic Zn/Co-ZIF as co-catalysts for photocatalytic CO_2_ reduction.^310^ Under visible light irradiation with [Ru(bpy)_3_]^2+^ as the photosensitizer and triethanolamine (TEOA) as the electron donor, C-BMZIFs (Zn/Co = 3/1) delivered a high CO yield of 1.1 × 10^4^ mmol g^−1^ h^−1^. Interestingly, decreasing the Zn/Co ratio in the Zn/Co-ZIF precursors could increase the size of Co particles in C-BMZIFs, which could favor the evolution of H_2_ against CO, resulting in the decrease of the CO/H_2_ ratio in the produced syngas. Other nanomaterials derived from bimetallic MOFs, such as CuNi/C^311^ and ZnO/NiO,^312^ have also been designed for photocatalytic reduction of CO_2_.

#### 5.1.3 Electrocatalysis

Functional nanomaterials derived from bimetallic MOFs have been employed as electrocatalysts for efficient and stable electrocatalysis of the ORR, OER, and HER, with applications in proton exchange membrane fuel cells (PEMFCs), alkaline fuel cells (AFCs), metal–air batteries and overall water splitting.^313–315^ Bimetallic MOF-derived nanomaterials have also been applied in electrochemical CO_2_/N_2_ reduction.

**Fig. 21:**
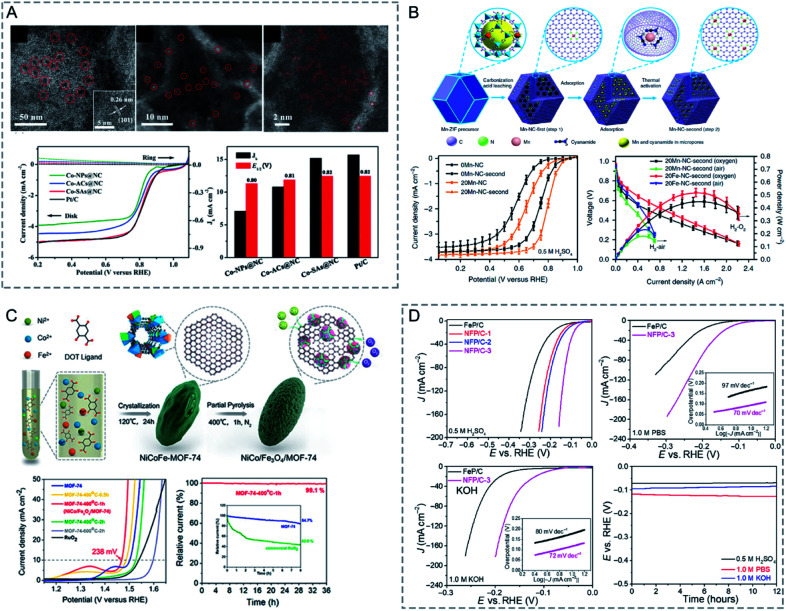
(A) TEM images of Co-NPs@NC (left top), HAADF-STEM images of Co-ACs@NC (middle top) and Co–SAs@NC (right top). ORR polarization curves obtained using RRDEs at 1600 rpm and *J*_k_ at 0.6 V and *E*_1/2_ for synthesized Co-NPs@NC, Co-ACs@NC, Co–SAs@NC and Pt/C catalysts (bottom). Reproduced with permission from ref. 318. Copyright 2019, Wiley-VCH. (B) Schematic of atomically dispersed MnN_4_ site catalyst synthesis (top). Steady-state ORR polarization plots in 0.5 M H_2_SO_4_ electrolytes (Pt/C catalyst reference was studied in 0.1 M HClO_4_) to study the effect of synthesis steps (that is, first doping and second adsorption) on Mn–N–C catalyst activity (left bottom). Fuel cell performance of the best-performing 20Mn-NC-second and 20Fe-NC-second catalysts under both H_2_/O_2_ and H_2_/air conditions. Error bars represent the standard deviation from at least three independent measurements (right bottom). Reproduced with permission from ref. 239. Copyright 2018, Nature Publishing Group. (C) Scheme of the fabrication of trimetal NiCoFe-MOF-74 and the partial pyrolysis of NiCoFe-MOF-74 to NiCo/Fe_3_O_4_/MOF-74 (top). OER polarization curves of NiCoFe-MOF-74 and the derived partial pyrolyzed and completely pyrolyzed samples at 1600 rpm (left bottom). Relative current of initial current density at a constant potential of 1.47 V as a function of test time (chronoamperometric method) (right bottom). NiCoFe-MOF-74-400 °C-1 h (NiCo/Fe_3_O_4_/MOF-74) (red curve), MOF-74 (blue curve), and commercial RuO_2_ (green curve). Reproduced with permission from ref. 265. Copyright 2018, American Chemical Society. (D) LSV curves of FeP/C and NFP/C-3 in 0.5 M H_2_SO_4_ and 1.0 M PBS and 1.0 M KOH solutions. Chronopotentiometry curves at a constant current density of 10 mA cm^−2^ for 12 hours of NFP/C-3 in 0.5 M H_2_SO_4_, 1.0 M PBS, and 1.0 M KOH solutions. Reproduced with permission from ref. 228. Copyright 2019, American Association for the Advancement of Science.

The ORR is the key reaction on the cathode of fuel cells and metal–air batteries. Bimetallic MOF derivatives,^244^ especially Fe and/or Co in N-doped carbon,^218,236,237,241,316^ have been investigated for ORR electrocatalysis.^317^ These supported metal catalysts, in the form of atomically dispersed metal sites, metal clusters, and metal NPs have demonstrated activities for the ORR. Deng and co-workers reported the controlled synthesis of NPs, atomic clusters (ACs), and SAs of Co catalysts on N-doped porous carbon derived from ZnCo-ZIFs for the ORR.^318^ Three samples with Zn/Co molar ratios of 0 : 1, 2 : 1, and 8 : 1 were synthesized, denoted as Co-ZIF, ZnCo-ZIF-2, and ZnCo-ZIF-8, respectively. Upon pyrolysis at 1173 K in Ar and subsequent reflux treatment in H_2_SO_4_ solution, Co-ZIF, ZnCo-ZIF-2, and ZnCo-ZIF-8 transformed into Co-NPs@NC, Co-ACs@NC, and Co–SAs@NC nanocomposites, respectively (Fig. 21A). These catalysts showed size-dependent ORR activity in a 0.1 M KOH electrolyte. Co–SAs@NC exhibited a superior activity with a half-wave potential (*E*_1/2_) of 0.82 V, outperforming those of Co-ACs@NC (0.81 V) and Co-NPs@NC (0.80 V). The determined electron transfer number of Co–SAs@NC is above 3.9, higher than those of Co-ACs@NC and Co-NPs@NC. These results demonstrated that isolated single Co atoms could provide maximum atom-utilization and be well stabilized by an N-doped carbon support to show superior reactivity and stability. Besides Fe and Co, Mn-based catalysts are promising alternatives to address the insufficient durability of ORR catalysts. Wang, Wu and co-workers synthesized an atomically dispersed Mn–N–C catalyst for the ORR, obtained by calcination of MnZn-ZIF-8 followed by adsorption and thermal activation processes to increase the density of MnN_4_ active sites (Fig. 21B).^239^ The 20Mn-NC-second catalyst (where 20 represents the molar percentage of Mn against the total metals for the synthesis of MnZn-ZIF-8; second refers to the second adsorption step) exhibited the most positive *E*_1/2_ of 0.8 V. Mn-NC-first showed poor ORR activity due to insufficient density of active sites. The 20Mn-NC-second catalyst showed a H_2_O_2_ yield of less than 2%, indicating a four-electron reduction pathway. The 20Mn-NC-second catalyst as a cathode in membrane electrode assemblies for fuel cells showed a power density of up to 0.46 W cm^−2^.

Efficient OER electrocatalysis is important for various energy-related processes, such as electrochemical water splitting and rechargeable metal–air batteries. Bimetallic MOF-derived metals/alloys embedded in carbon,^319^ metal compounds^248,261^ and their carbon composites^233,320–322^ have demonstrated OER activity.^323,324^ Xu and co-workers applied FeCo–P/C nanocomposites derived from bimetallic MOFs for the OER.^232^ Fe_*x*_Co_*y*_ bimetallic MOFs with various Fe/Co ratios (*x* = 1, 2 and *y* = 1, 2) were employed as precursors to synthesize Fe_*x*_Co_*y*_-P/C. The overpotentials of Fe_*x*_Co_*y*_-P/C in 1.0 M KOH at a current density of 10 mA cm^−2^ showed a trend of Fe_1_Co_1_–P/C (360 mV) < Fe_1_Co_2_–P/C (362 mV) < Fe_2_Co_1_–P/C (368 mV). All the Fe_*x*_Co_*y*_-P/C materials exhibited a better OER activity than their Fe_*x*_Co_*y*_/C without P-doping. The authors demonstrated that both P- and Fe-doping could reduce the resistance of charge transfer to accelerate the electron transfer and thus improve the OER activity. Considering that the complete decomposition of MOFs usually leads to a dramatic decrease of the surface area and pore structure, partial decomposition of bimetallic MOFs can preserve the porous structure to a certain extent for effective substrate diffusion while producing active metal NPs. Li and co-workers reported the synthesis of NiCo/Fe_3_O_4_/MOF-74 through controlled pyrolysis of trimetallic NiCoFe-MOF-74 for efficient OER electrocatalysis (Fig. 21C).^265^ The obtained NiCo/Fe_3_O_4_/MOF-74 retained 68% of the specific surface area of the pristine NiCoFe-MOF-74 (820 m^2^ g^−1^). In NiCo/Fe_3_O_4_/MOF-74, NiCo/Fe_3_O_4_ heterostructures with a NiCo alloy enriched in the core and Fe_3_O_4_ distributed on the shell were well dispersed in the partially retained MOF-74 structure. In a 1.0 M KOH electrolyte, NiCo/Fe_3_O_4_/MOF-74 exhibited an overpotential of 238 mV at 10 mA cm^−2^ on a glassy carbon electrode, surpassing those of NiCoFe-MOF-74 (270 mV), the totally decomposed NiCoFe-MOF-74 derivative (366 mV) and the commercial RuO_2_ catalyst (310 mV). NiCo/Fe_3_O_4_/MOF-74 could retain 99.1% of its initial OER activity after 36 h. The excellent activity and stability of NiCoFe-MOF-74 for the OER could be attributed to the porous structures of MOF-74 and the synergistic effect between NiCo and Fe_3_O_4_.

Efficient and inexpensive HER electrocatalysis to produce hydrogen from water splitting is of significant importance. Bimetallic MOF-derived materials, especially Mo-based materials^325^ and metal nitrides/phosphides/sulphides,^253,254,258,259,326^ have been intensively investigated due to their favourable hydrogen adsorption energy toward the HER.^327^ Lou and co-workers synthesized carbon-supported Ni-doped FeP nanocrystals from Ni-doped MIL-88A as HER electrocatalysts.^228^ Ni-doped MIL-88A was treated with phytic acid and subsequent pyrolysis under Ar and H_2_ to obtain Ni-doped FeP/C. The overpotential at 10 mA cm^−2^ of NFP/C-3 (Ni/Fe atomic ratio of 3) was 72, 117, and 95 mV in 0.5 M H_2_SO_4_ (pH = 0), 1.0 M phosphate-buffered saline (pH ≈ 7) and 1.0 M KOH (pH = 14), respectively (Fig. 21D). The NFP/C-3 electrocatalyst also exhibited good stability over the full pH range. The carbon support is important to structural integrity and catalytic performance. NFP-3 without carbon showed degradation of electrocatalytic activity and stability. XPS analysis and DFT calculations demonstrated that Ni doping could weaken the strong hydrogen adsorption on pure FeP to favour the HER.

**Fig. 22:**
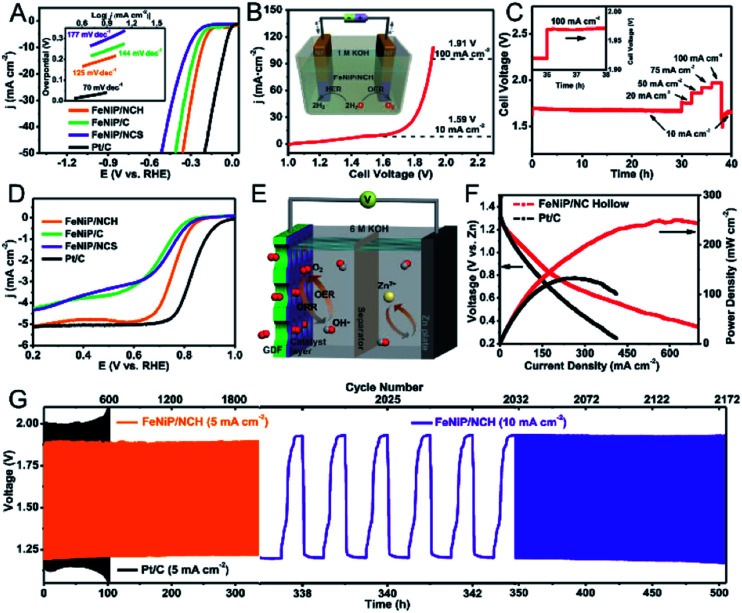
(A) HER polarization of FeNiP/NCH and control samples in 1 M KOH at a scan rate of 10 mV s^−1^. Inset: corresponding Tafel curves. (B) Polarization curve of the FeNiP/NCH‖FeNiP/NCH electrode in a two-electrode configuration for water splitting. Inset: representation of the water-splitting device. (D) Catalytic stability at different current densities for around 40 h. Inset: the enlarged region of the curves for 100 mA cm^−2^. Notably, in each increment of current density, the voltage could increase accordingly and then stabilize rapidly, whereafter, the cell voltage was also completely restored when the current density finally decreased to 10 mA cm^−2^. (D) ORR polarization of FeNiP/NCH and control samples in O_2_-saturated 0.1 M KOH solution with a RDE rotation rate of 1600 rpm. (E) Representation of a Zn–air battery (GDF: gas diffusion film). (F) Battery voltage and power density of Zn–air batteries. (G) Galvanostatic discharge–charge cycling curves of rechargeable Zn–air batteries with FeNiP/NCH and Pt/C catalysts on Ni foam, respectively. Reproduced with permission from ref. 94. Copyright 2019, American Chemical Society.

Highly active and robust bimetallic MOF-derived multifunctional electrocatalysts for the ORR, OER and/or HER can be applied for efficient rechargeable metal–air batteries^185,219,224,315^ and overall water splitting.^230,233,258,328–330^ Very recently, Xu and co-workers synthesized a capsular carbon embedded with iron-nickel phosphide NPs (FeNiP/NCH) through pyrolysis of capsular-MOF followed by phosphidation with melamine.^94^ FeNiP/NCH was employed as a trifunctional electrocatalyst for the ORR/OER/HER. In 1.0 M KOH solution, FeNiP/NCH obtained a current density of 10 mA cm^−2^ at overpotentials of 250 and 216 mV for the OER and HER, respectively. For ORR electrocatalysis in 0.1 M KOH solution, FeNiP/NCH exhibited an *E*_1/2_ of 0.75 V. When FeNiP/NCH was applied as both cathode and anode electrocatalysts for overall water splitting, a small cell voltage of 1.59 V was achieved at 10 mA cm^−2^ (Fig. 22). When FeNiP/NCH was applied as the air electrode electrocatalyst in a Zn–air battery, a high power density (250 mW cm^−2^) could be achieved. In a galvanostatic discharge–charge cycling test at 5 mA cm^−2^, the charge/discharge potentials were 1.89 and 1.23 V with a small voltage gap of 0.66 V. Moreover, no obvious voltage gap change was observed for 500 h, indicating the high stability of FeNiP/NCH.

Electrochemical CO_2_ reduction is a potentially effective approach to produce synthetic fuels utilizing waste CO_2_.^331^ Bimetallic MOF-derived nanomaterials demonstrated impressive FE for CO_2_ reduction.^238,240,332,333^ Pan, Liu, Jiang and co-workers employed single-atom Ni implanted N doped carbon catalysts (Ni_SA_–N_*x*_–C) with a controlled N coordination number (*x* = 2, 3, 4) derived from a MgNi-MOF-74 confined polypyrrole (PPy) composite for the electrochemical reduction of CO_2_ to CO.^240^ The CO FE of Ni_SA_–N_*x*_–C followed a trend of Ni_SA_–N_2_–C > Ni_SA_–N_3_–C > Ni_SA_–N_4_–C. The TOF values for CO production of Ni_SA_–N_2_–C, Ni_SA_–N_3_–C and Ni_SA_–N_4_–C are 1622, 1120, and 225 h^−1^ at −0.8 V, respectively. Ni_SA_–N_2_–C possessed the highest CO_2_ reduction activity among the Ni_SA_–N_*x*_–C catalysts, attributed to the favorable formation of the COOH* intermediate on low coordinated Ni–N_2_ sites. Sun, Han and co-workers applied MOF-derived In-Cu bimetallic oxide catalysts (InCuO-*x*, where *x* is the Cu/In molar ratio) for CO_2_ electroreduction.^332^ InCuO-0.92 showed a highest FE of 92.1% at a potential of −0.8 V, which was 1.1, 1.3, 1.7, and 3.0 times higher than that of InCuO-0.72, InCuO-0.55, InCuO-0.37, and InCuO-0.15, respectively. The increase of *x* led to enhanced CO_2_ reduction activity, indicating the synergistic effect between In oxides and Cu oxides to lead to stronger CO_2_ adsorption, a higher electrochemical surface area and lower charge transfer resistance.

Electrochemical N_2_ reduction (NNR) under ambient conditions is a promising sustainable route for ammonia synthesis.^334^ The related study on bimetallic MOF derivatives for NNR is still in its infancy. Recently, Qin, Cho and co-workers synthesized MoFe embedded in phosphorus-doped carbon microspheres (MoFe-PC) derived from bimetallic MoFe-MOFs for electrocatalytic NNR.^335^ The MoFe-PC catalyst gave a NH_3_ formation rate of 34.23 μg h^−1^ mg_cat._^−1^ with a high FE of 16.83% at −0.5 V, surpassing those of Mo–C (12.52 μg h^−1^ mg_cat._^−1^, FE: 9.67%), Fe–C (17.83 μg h^−1^ mg_cat._^−1^, FE: 7.69%), and MoFe–C (24.73 μg h^−1^ mg_cat._^−1^, FE: 12.47%) prepared from pyrolysis of Mo-MOF, Fe-MOF, and MoFe-MOF without phosphorization, respectively. The superior performance of MoFe-PC for NNR can be attributed to the synergistic effect of Mo and Fe oxides and P-doped carbon.

### 5.2 Energy storage and conversion

Bimetallic MOF-derived nanomaterials have attracted significant attention for energy storage applications such as supercapacitors and rechargeable batteries.^336–339^

#### 5.2.1 Supercapacitors

Bimetallic compounds (hydroxides,^250,251^ oxides,^245,340,341^ and sulphides^193,255^) and their carbon composites,^342–344^ as well as metal@carbon composites^345–347^ have been employed as electrode materials for supercapacitors.

**Fig. 23:**
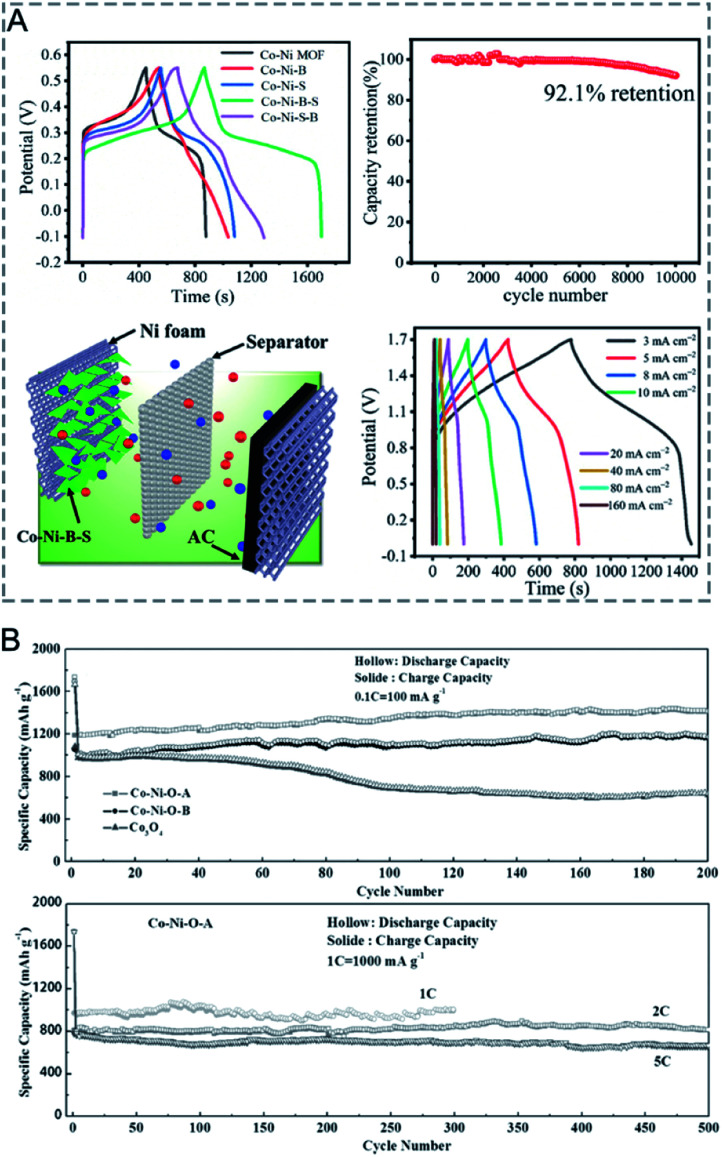
(A) GCD plots of the prepared samples and cycling life of Co–Ni–B–S at 15 A g^−1^ (top). Schematic illustration of the asymmetric Co–Ni–B–S/NF//AC/NF device and GCD profiles at various current densities in the voltage range of 0 to 1.7 V (bottom). Reproduced with permission from ref. 255. Copyright 2019, Wiley-VCH. (B) Cycling performances of various products of Co–Ni–O–A, Co–Ni–O–B, and Co_3_O_4_ at 100 mA g^−1^ (0.1C) and high-rate cycling performances of Co–Ni–O–A. Reproduced with permission from ref. 243. Copyright 2015, Wiley-VCH.

Superior to monometallic compounds, bimetallic compounds can enable richer redox reactions and higher electrical conductivity owing to charge transfer between different ions. Bimetallic compounds with tuneable compositions reveal facile and reversible faradaic behaviors for excellent electrochemical capacitors. Bimetal oxides derived from bimetallic MOFs have high specific capacities but may suffer from unsatisfactory conductivity. Converting bimetallic MOFs into phosphides and sulphides can improve the electrical conductivity. For example, Xia and co-workers applied cobalt/nickel boride/sulphide (Co–Ni–B–S) derived from bimetallic Co–Ni MOFs for supercapacitors.^255^ A Co–Ni MOF was first treated with sodium borohydride to lower the valence state of Co/Ni species (Co–Ni–B), followed by a sulfurization process to yield Co–Ni–B–S. When tested for supercapacitors, the Co–Ni–B–S electrode exhibited a high specific capacitance of 1281 F g^−1^ at 1 A g^−1^, with a high retention of 92.1% after 10 000 cycles (Fig. 23A). The energy density of the Co–Ni–B–S electrode could reach as high as 50.0 Wh kg^−1^ at a power density of 857.7 W kg^−1^, with a capacity retention of 87.7% at a 12 A g^−1^ after 5000 cycles.

Metal compounds with complex structures can induce abundant hetero-interfaces and tune the electronic structure to facilitate ion/electron transfer and improve the specific capacity.^245,252,348^ Hu, Lou and co-workers fabricated CoO/Co–Cu–S hierarchical tubular heterostructures (HTHSs) for hybrid supercapacitors.^348^ CoO/Co–Cu–S was obtained by calcination of polyacrylonitrile (PAN)@MOF-74(Co/Cu) in air followed by annealing with S powder in a N_2_ atmosphere. CoO/Co–Cu–S-2 (2 indicates the molar ratio of Co^2+^/Cu^2+^ in the precursors) delivered a specific capacity of 320 mA h g^−1^ at a current density of 2.0 A g^−1^, higher than that of CoO/Co–Cu–S-1 (192 mA h g^−1^), CoO/Co–Cu–S-0.5 (167 mA h g^−1^), CoO/Co–Cu–S-2 nanoneedles without a tubular structure (135 mA h g^−1^), monometallic CoO/CoS_*x*_ nanofibers (110 mA h g^−1^) and Cu_1.81_S nanofibers (97 mA h g^−1^). A hybrid supercapacitor constructed with CoO/Co–Cu–S-2 and activated carbon electrodes showed a high and stable energy density of 90.7 Wh kg^−1^ at a power density of 800 W kg^−1^.

Constructing metal compound and carbon composites can improve the electrical conductivity, accommodate the volumetric change, and prevent the aggregation and dissolution of the metal compounds, thereby improving the rate capability and cycling stability. Cai, Zhang and co-workers synthesized bimetallic nickel cobalt sulphide embedded nitrogen-doped carbon composites (Ni–Co–S-*n*/NC, *n* represents the Ni/Co molar ratio) with a hollow spherical structure for supercapacitors.^344^ The Ni–Co–S-0.5/NC composite showed high specific capacities of 543.9 and 366.0C g^−1^ at 1 and 20 A g^−1^, respectively. An asymmetric supercapacitor based on the hollow Ni–Co–S-0.5/NC composite as the cathode exhibited good electrochemical performance with a high energy density of 39.6 W h kg^−1^ at a power density of 808 W kg^−1^.

#### 5.2.2 Batteries

Materials derived from bimetallic MOFs have been investigated in a variety of batteries, including LIBs,^349^ sodium-ion batteries (SIBs),^350^ LOBs,^351^*etc.*

For LIBs, bimetallic oxides with complex chemical compositions have been designed to provide enhanced electrical conductivity and rich redox-active sites.^243,352–354^ Sun, Wang and co-workers used Ni_*x*_Co_3−*x*_O_4_ nanorods derived from Co/Ni-MOF-74 for lithium storage.^243^ Co/Ni-MOF-74 A (Co : Ni = 9 : 1) and Co/Ni-MOF-74 B (Co : Ni = 2 : 1) afforded Ni_0.3_Co_2.7_O_4_ and NiCo_2_O_4_ after calcination at 450 °C in air, respectively. Ni_0.3_Co_2.7_O_4_ nanorods exhibited a large reversible Li-storage capacity of 1410 mA h g^−1^ after 200 cycles at 100 mA g^−1^, which was higher than that of NiCo_2_O_4_ (1157 mA h g^−1^) and Co_3_O_4_ (625 mA h g^−1^). Ni_0.3_Co_2.7_O_4_ also showed large reversible capacities of 812 and 656 mA h g^−1^ after 500 cycles at large current densities of 2 and 5 A g^−1^, respectively (Fig. 23B). The excellent performances of Ni_0.3_Co_2.7_O_4_ could be attributed to the mesoporous nanorod structure and the synergistic effect of two active metal oxides. Hybrid metal oxides with complex structures, such as ZnO/ZnFe_2_O_4_ sub-microcubes,^355^ Fe_2_O_3_@NiCo_2_O_4_ nanocages,^246^ and CuO@NiO hollow spheres,^247^ have been designed to accommodate large volume variation and shorten the lithium-ion diffusion length.

Embedding bimetal oxides in porous carbon can accommodate volume change and enhance electron/ion transport to greatly enhance the lithium storage performance. In this regard, bimetallic MOF-derived bimetallic oxide/carbon composites, such as CuCo_2_O_4_/C,^356^ ZnFe_2_O_4_/C@N-doped carbon nanotubes,^357^ and ZnO/ZnFe_2_O_4_/C,^358^ have been designed as anode materials for LIBs. In addition, other bimetal alloys and compounds (sulphides and phosphides) supported on carbon composites^234,235,344,345,359,360^ derived from bimetallic MOFs were also reported as anode materials for LIBs.

MOF-derived bimetallic compounds and their carbon composites can also be applied as anodes for SIBs. Bimetallic MOF-derived metal compounds, such as CoFe_2_O_4_ (ref. 361) and Co_3_O_4_/ZnO,^362^ could deliver high capacities but may suffer from poor rate capability and cycling stability due to a low electrical conductivity and large volume variation during charge/discharge processes. Bimetallic MOF-derived metal compound/carbon composites such as (Co_0.5_Ni_0.5_)_9_S_8_/N–C^231^ and Ti-doped-CoO@C,^363^ have been demonstrated as high-performance anode materials for SIBs. The porous carbon components can facilitate electron/ion transport and improve the structural integrity, and the metal compound components can deliver high capacities for sodium storage.

Bimetallic MOF-derived materials have also been applied as cathode materials for LOBs. Bimetallic MOF-derived metal compounds and their carbon composites, such as Co–Mn–O nanocubes,^352^ Co_3_O_4_@graphitic porous carbon,^364^ and ZnO/ZnFe_2_O_4_/C nanocages,^365^ have showed high performance in LOBs. In metal compound/carbon composites, the carbon supports with a large pore volume are beneficial for mass transportation and accommodation of discharge products (*e.g.*, LiO_2_), and the well distributed catalysts accelerate oxygen-related redox reactions.

## 6. Conclusions and prospects

Bimetallic MOFs increase the complexity of MOF materials. Bimetallic MOFs can be divided into two categories according to the metal distribution, namely, solid solution and core–shell bimetallic MOFs. In solid solution bimetallic MOFs, two different metal ions have delocalized or even homogeneous distributions throughout the whole MOF crystals. Direct synthesis, post-synthetic modifications, and template synthesis have been applied for the preparation of solid solution bimetallic MOFs. A variety of techniques should be combined to well characterize the composition, location, and arrangement of metals in the synthesized bimetallic MOFs. Many studies lack detailed characterization to identify the metal arrangements within the frameworks, either mixed in the same SBUs or separated in different SBUs. The identification of the metal arrangements in bimetallic MOFs is of vital importance to correlate the metal mixing patterns with properties.

Two MOFs with different metal centres can be assembled into core–shell bimetallic MOFs. Seed-induced growth, post-synthetic exchange, and one-pot synthesis have been developed for the synthesis of core–shell bimetallic MOFs. The assembly of core–shell structures usually leads to distorted interfaces with defects and modified crystal structures and thus induces new mechanical, electronic, and catalytic properties. In future work, detailed characterization of the interfaces is expected, which will improve the fundamental understanding of the assembly process and establish structure–property relationships.

Bimetallic MOFs with complex compositions and structures frequently show superior properties to their monometallic counterparts. Metal substitution in the SBUs can tune the stability, flexibility, pore structure, and electronic structure of bimetallic MOFs. The possibility to tune the physical and chemical properties provides bimetallic MOFs with great promise in many applications, including gas adsorption, catalysis, energy storage and conversion, and luminescence sensing. Moreover, bimetallic MOFs with well-defined crystal structures are suitable for atomically precise structural characterization and computational modelling to achieve fundamental understanding of the structure–performance relationship. Despite these advantages, the industrial applications of bimetallic MOFs are restricted due to their low stability, microporous structure, blockage of active sites, and poor conductivity.

Especially, the stability issue of bimetallic MOFs should be addressed if they are to be applied in industrial applications. Much effort has been devoted to improving the stability of MOFs, including direct synthesis of high-valent metal-carboxylate or low-valent metal-azolate MOFs and post-synthetic modification of organic linkers or coating of a protective layer. General and facile strategies are to be developed to be applicable to different bimetallic MOF systems. It is necessary to systematically investigate the chemical, thermal, and mechanical stability of bimetallic MOFs to guide the synthesis of very stable bimetallic MOFs for targeted applications.

Moreover, bimetallic MOFs can serve as excellent precursors/templates for the synthesis of a variety of nanostructured materials, including carbon composites, metal compounds, and MOF composites. The achieved success in controlling the compositions and distributions of metal ions in bimetallic MOFs can allow the tuning of compositions and structures of the obtained metal atoms/NPs/compounds. The homogeneous doping of heteroatoms (N, P, S, *etc.*) in the organic linkers can be inherited to the derived carbon materials to stabilize the metal species and facilitate the activity. The different coordination abilities of metal ions with the organic linkers in bimetallic MOFs offer the possibility to selectively transform part of the framework to metal NPs/compounds while preserving the rest of the framework to retain the porosity to a certain extent.

Bimetallic MOF derivatives show exposed active sites, good stability and conductivity, enabling them to extend their applications in the catalysis of more challenging reactions under harsh conditions and electrochemical energy storage and conversion. Despite the achieved success in the transformation of bimetallic MOFs to functional materials, several common drawbacks persist during the transformation process: (1) the poor control over the pore structure of bimetallic MOF-derived materials and (2) the inevitable aggregation of metal NPs/compounds. To address these issues, template-assisted synthesis and controlled transformation to “quasi-MOFs” are currently emerging to boost the development of bimetallic MOF-derived nanomaterials. Template-assisted synthesis utilizing SiO_2_, *etc.*, can provide robust skeletons to prevent the architecture from collapsing and serious aggregation of metal NPs/compounds during calcination. Besides, controlled transformation into quasi-MOFs can not only allow the exposure of active sites, but also preserve the porous structures to a certain extent. The unique features of quasi-MOFs remain to be explored, which will pave the way to the development of the next-generation of functional materials.

Although many challenges still exist, the rapid development of bimetallic MOFs and their derivatives in recent years will lead to a promising future. Continued research and development in this exciting area can be expected to enable the practical applications of bimetallic MOF-based materials.

## Conflicts of interest

There are no conflicts to declare.
